# Effects of Site-Specific Glycation on α‑Synuclein

**DOI:** 10.1021/acschembio.6c00322

**Published:** 2026-06-18

**Authors:** Tim Baldensperger, Anna Hampel, Christian F. W. Becker

**Affiliations:** † Institute of Biological Chemistry, Faculty of Chemistry, 27258 University of Vienna, Währinger Straße 38, 1090 Vienna, Austria; ‡ Research Platform ‘Non-Enzymatic Protein Modifications in Neurodegeneration’, 27258 University of Vienna, Währinger Straße 38, 1090 Vienna, Austria

## Abstract

Glycation of α-synuclein (αSyn) by methylglyoxal (MGO) has been implicated as a pathologic mechanism in Parkinson’s disease. However, mechanistic understanding has been limited so far by the heterogeneity of chemically MGO-modified αSyn. We developed and applied two complementary semisynthetic strategies based on native chemical ligation for site-specific incorporation of *N* ^ε^-carboxyethyllysine (CEL), a major advanced glycation endproduct formed by MGO. Using this approach, we generated a panel of 11 αSyn variants bearing CEL modifications at defined positions in the *N*-terminal region. Single CEL modifications did not alter the intrinsically disordered nature of αSyn, but significantly reduced membrane-induced folding upon interaction with anionic phospholipid vesicles. Aggregation analyses using dynamic light scattering, thioflavin T fluorescence, and sedimentation assays revealed stabilization of small αSyn oligomers and attenuation of fibril formation. Moreover, distinct changes in aggregate morphology were induced by specific CEL modifications. Consistent with these effects, several CEL-modified αSyn variants exhibited a site-dependent reduction in seeding capacity compared to wild-type aggregates, with K10CEL as a notable exception that retained seeding activity. In summary, our results demonstrate that single CEL modifications efficiently modulate αSyn function and aggregation. The semisynthetic platform established here enables elucidation of glycation effects on αSyn and provides a general framework for studying the effects of AGEs in synucleinopathies.

## Graphical abstract

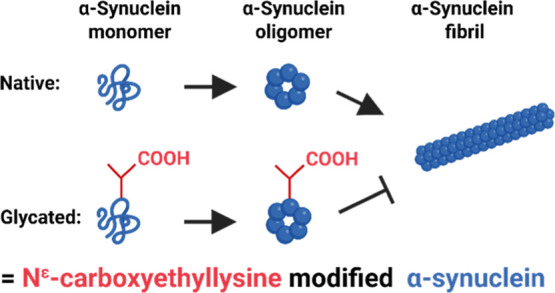

## Introduction

Parkinson’s disease (PD) was first described by James Parkinson in 1817 as a neurodegenerative disorder characterized by motor symptoms such as tremor, bradykinesia, and rigidity.^1^ PD is the fastest growing neurological pathology with more than 11 million patients globally.^2,3^ Despite extensive research during the last few decades, an efficient treatment to cure PD is still unavailable.^4^

Central hallmarks of PD are the progressive loss of dopaminergic neurons and the accumulation of α-synuclein (αSyn) aggregates.^5^ Under physiological conditions, the intrinsically disordered protein αSyn is predominantly localized to presynaptic terminals, where it regulates synaptic vesicle trafficking and neurotransmitter release.^6^ This critical function of αSyn is enabled by multiple imperfect repeats of the lysine-rich KTKEGV motif in the *N*-terminal region (Figure 1A), which facilitate electrostatic interactions with negatively charged phospholipid membranes.^7^ Upon membrane binding, αSyn undergoes conformational changes and adopts partially α-helical structures.^8^ In contrast, the central non-amyloid β-component (NAC) region drives aggregation by promoting β-sheet formation.^9^ In synucleinopathies such as PD, monomeric αSyn adopts β-sheet-rich structures that assemble into oligomers, protofibrils, and finally insoluble fibrils.^10^ Increasing evidence suggests that especially small αSyn oligomers, rather than large fibrils, are key mediators of disease propagation and neurotoxicity.^11^

**Figure 1:**
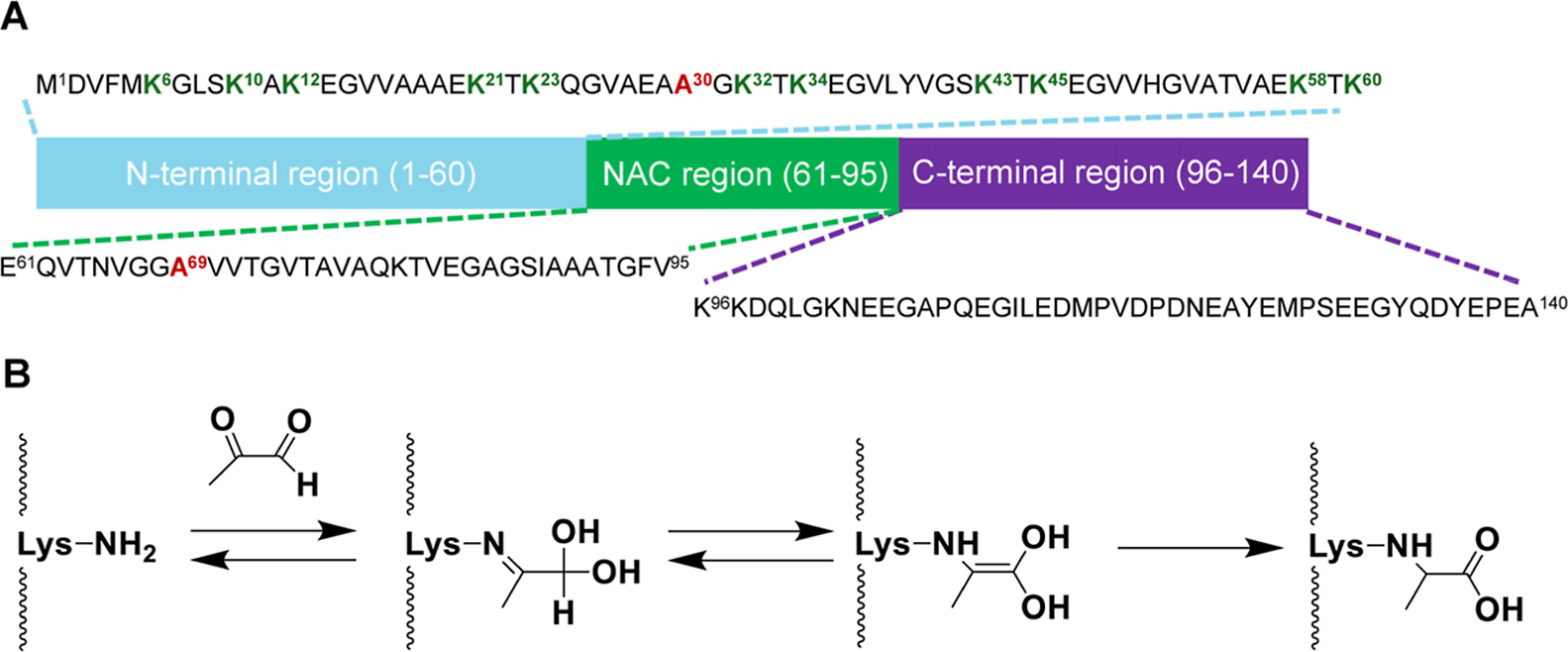
Glycation of αSyn. (A) Schematic structure and amino acid sequence of αSyn. The 11 *N*-terminal residues modified in this study are marked in green and ligation sites are marked in red. (B) Formation of N^ε^-carboxyethyllysine (CEL) via modification of lysine residues by methylglyoxal (MGO).

Only 5%–10% of PD patients have a genetic predisposition and the majority of PD cases occur sporadic.^12^ PD prevalence increases sharply after 60 years of age, making aging the dominant risk factor.^13^ Additional risk factors include exposure to environmental toxins, head injuries, and certain comorbidities.^14^ Notably, type 2 diabetes is associated with accelerated disease progression and increases PD risk by 1.1–2.8-fold, suggesting shared pathogenic mechanisms.^15,16^ Many of these risk factors promote chronic metabolic stress by generating excessive reactive oxygen species and reactive carbonyl species.^17^ These reactive metabolites lead to non-enzymatic protein modifications (nPTMs), which have been implicated as modulators of the αSyn structure, aggregation, and neurotoxicity.^18^

Increased triosephosphate metabolism in diabetes leads to elevated levels of the highly reactive 1,2-dicarbonyl methylglyoxal (MGO).^19^ MGO reacts with proteins to form advanced glycation endproducts (AGEs),^20^ among which N^ε^-carboxyethyllysine (CEL) is particularly abundant and accumulates during aging.^21^ In analogy to N^ε^-carboxymethyllysine (CML) formation by glyoxal,^22^ CEL is formed by an intramolecular Cannizzaro reaction (Figure 1B).^23^ Previous studies have shown that MGO-mediated glycation primarily affects the *N*-terminal region of αSyn and that CEL is enriched in PD patient brains.^24^ Moreover, a single intracerebroventricular injection of MGO exacerbated PD-like symptoms in mice^25^ and glycation of αSyn by MGO in vitro drastically reduced vesicle binding affinity^26^ and inhibited fibrillization.^27^

While these findings highlight the potential role of glycation in modulating αSyn pathobiology, they are based on global MGO treatment, affecting numerous cellular targets and generating heterogeneous αSyn carrying a variable number of nPTMs at multiple sites.^19,28^ This lack of specificity complicates the mechanistic interpretation of glycation effects on αSyn. To overcome this limitation, we utilize a flexible protein semisynthesis approach that allows site-selective modification of αSyn. Similar strategies were previously applied for αSyn synthesis to study the effects of defined PTMs such as acetylation, glycosylation (O-GlcNAc), phosphorylation, and nitration.^29–32^ By adopting this approach, we generated αSyn variants with site-specific CEL modifications in the *N*-terminal region, which is the preferred region for MGO-induced glycation.^24^ These chemically defined αSyn variants enabled systematic investigation of glycation effects on vesicle binding, aggregation, and seeding capacity.

## Results and Discussion

### CEL-Modified αSyn Variants via a Two-Segment Ligation Strategy

To achieve site-specific incorporation of CEL at positions 6, 10, 12, 21, and 23, we utilized a two-segment semisynthesis strategy that links an *N*-terminal synthetic peptide (aa 1–29) with a recombinantly expressed segment (aa 30–140) (Figure 2A). This approach provided the basis for investigating the effects of site-specific glycation on αSyn pathology.

**Figure 2:**
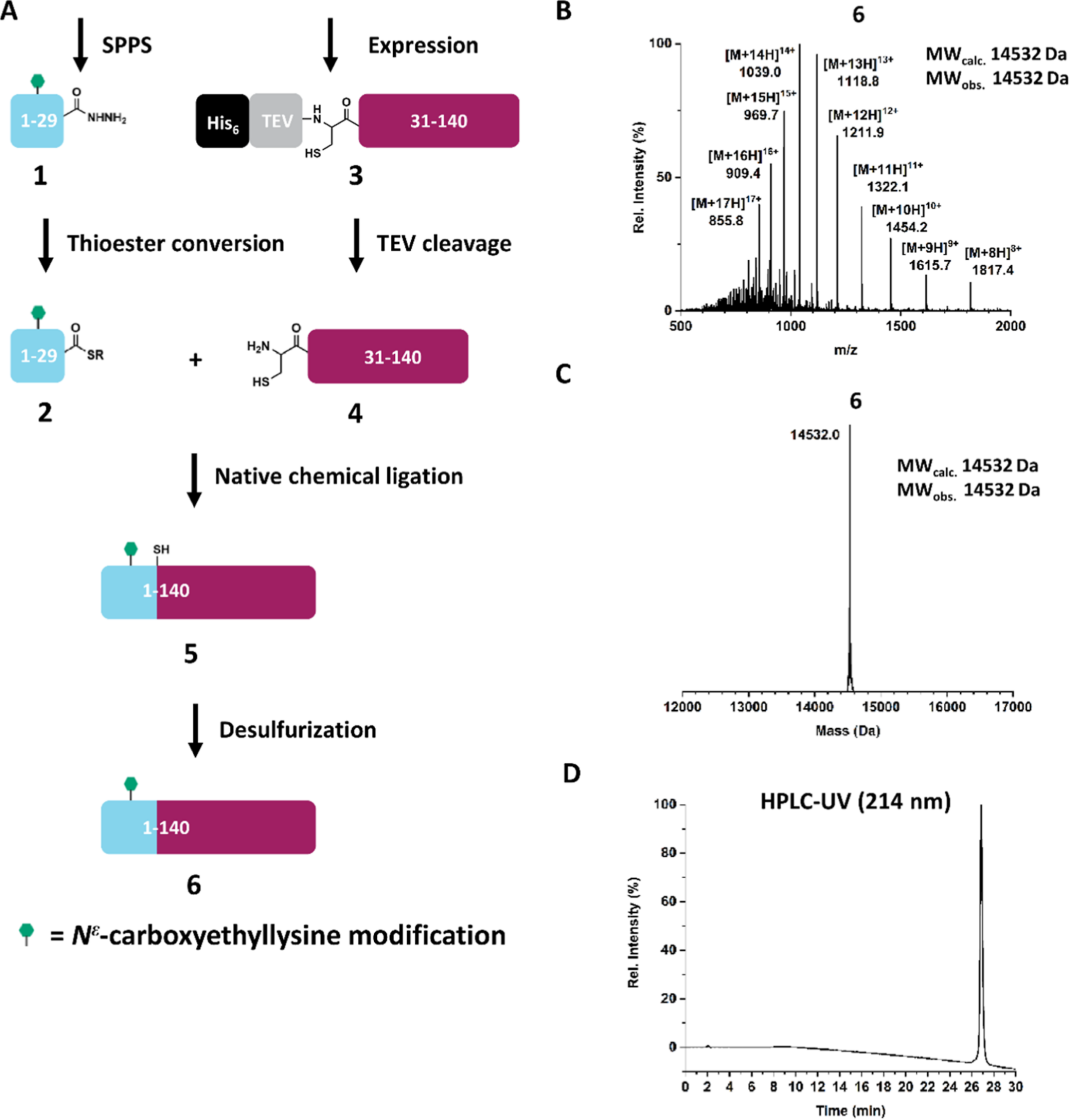
Two-segment ligation approach of αSyn. (A) Schematic overview of the semisynthetic strategy to generate CEL-modified αSyn at positions 6, 10, 12, 21, and 23. (B) Mass spectrum and (C) deconvoluted spectrum of CEL-modified full-length αSyn **6**. (D) Purity of **6** was determined by HPLC-UV at 214 nm absorbance.

First, *N*-terminal αSyn 1–29 segment **1** was synthesized as a peptide hydrazide via Fmoc solid-phase peptide synthesis (SPPS). This technique enabled selective replacement of defined lysine residues with CEL through coupling of a commercially available CEL building block. The successful synthesis of **1** was confirmed by mass spectrometry (MS) analysis (Figure S1A) and gave yields of 23%–45%, depending on the position of CEL incorporation. Conversion of peptide hydrazide **1** to sodium 2-mercaptoethanesulfonate (MESNa) thioester **2** (Figure S1B) proceeded with high yields of 71%–88%, which is in full agreement with well-established protocols.^33,34^ The purity of **2** was verified by HPLC-UV (Figure S1C).

In contrast to earlier multistep semisyntheses of αSyn,^33,35^ the remaining C-terminal portion was expressed recombinantly to overcome limitations of sequence length in SPPS and to minimize the number of ligation steps. Fusion construct **3** (His_6_-TEV-αSyn 30-140) was expressed in *E. coli* and the *N*-terminal His_6_-tag was used for efficient purification (35 mg/L expression medium) by Ni-NTA affinity chromatography (Figure S1D). Purified **3** was processed by tobacco etch virus (TEV) protease, cleaving the TEV recognition motif ENLYFQC between glutamine and cysteine with 78% yield, similar to previous reports.^34^ Formation of segment **4** (αSyn 30-140 A30C) was verified by MS (Figure S1E), and purity was confirmed by HPLC-UV (Figure S1F). In addition to removing the His_6_-tag, TEV cleavage released the *N*-terminal cysteine residue, which is required for the following native chemical ligation (NCL) reaction.

NCL between *N*-terminal peptide thioester **2** and recombinant segment **4** was performed under denaturing conditions in guanidine hydrochloride (GdnHCl), and MPAA was used as a thiol additive.^36^ Segment **4** was used at a 1 mM concentration and **2** was added at a 2-fold molar excess to compensate for possible thioester hydrolysis. Together, these conditions enabled efficient ligation, resulting in 59%–69% isolated yields of CEL-modified ligation product **5**, whose identity was confirmed by MS (Figure S1G). Radical-mediated desulfurization by VA-044 and TCEP restored the native alanine at position 30.^37^ The observed mass of desulfurized product **6** matched the calculated mass of 14,532 Da (Figure 2B,C), and no impurities were detected by reverse-phase high-performance liquid chromatography (RP-HPLC) analysis (Figure 2D). Desulfurization proceeded quantitatively and isolated yields of 72%–81% were in good agreement with previous semisyntheses of αSyn.^33,35^

In summary, the combination of SPPS on a 0.05 mmol scale with recombinant expression in 2 L of culture medium was sufficient to generate 10–20 mg of each CEL-modified αSyn variant. Combined ligation and desulfurization yields were 48%–55%, which is a clear improvement compared to previously reported four-segment ligation with 10% total yield.^33^ To benchmark the efficiency of the two-segment NCL approach and generate a suitable control for further experiments, unmodified αSyn WT was synthesized using the same strategy. Isolated yields were within ±5%–10% of those obtained for the CEL-modified variants, and comparable purity was achieved (Figure S2).

### CEL-Modified αSyn Variants via a Three-Segment Ligation Strategy

Incorporation of CEL at more central residues within the *N*-terminal region such as positions 32, 34, 43, 45, 58, and 60 required the development of a three-segment NCL strategy (Figure 3A). Again, this approach made use of the segment αSyn 1–29, which was synthesized in contrast to the two-segment strategy without CEL-modification as peptide hydrazide **7** (Figure S3A). Successful conversion to thioester **8** and purity were detected by MS and HPLC-UV, respectively (Figure S3B,C). The remaining αSyn sequence was subdivided into a central CEL-modified segment generated by SPPS and a C-terminal fragment that was expressed recombinantly.

**Figure 3:**
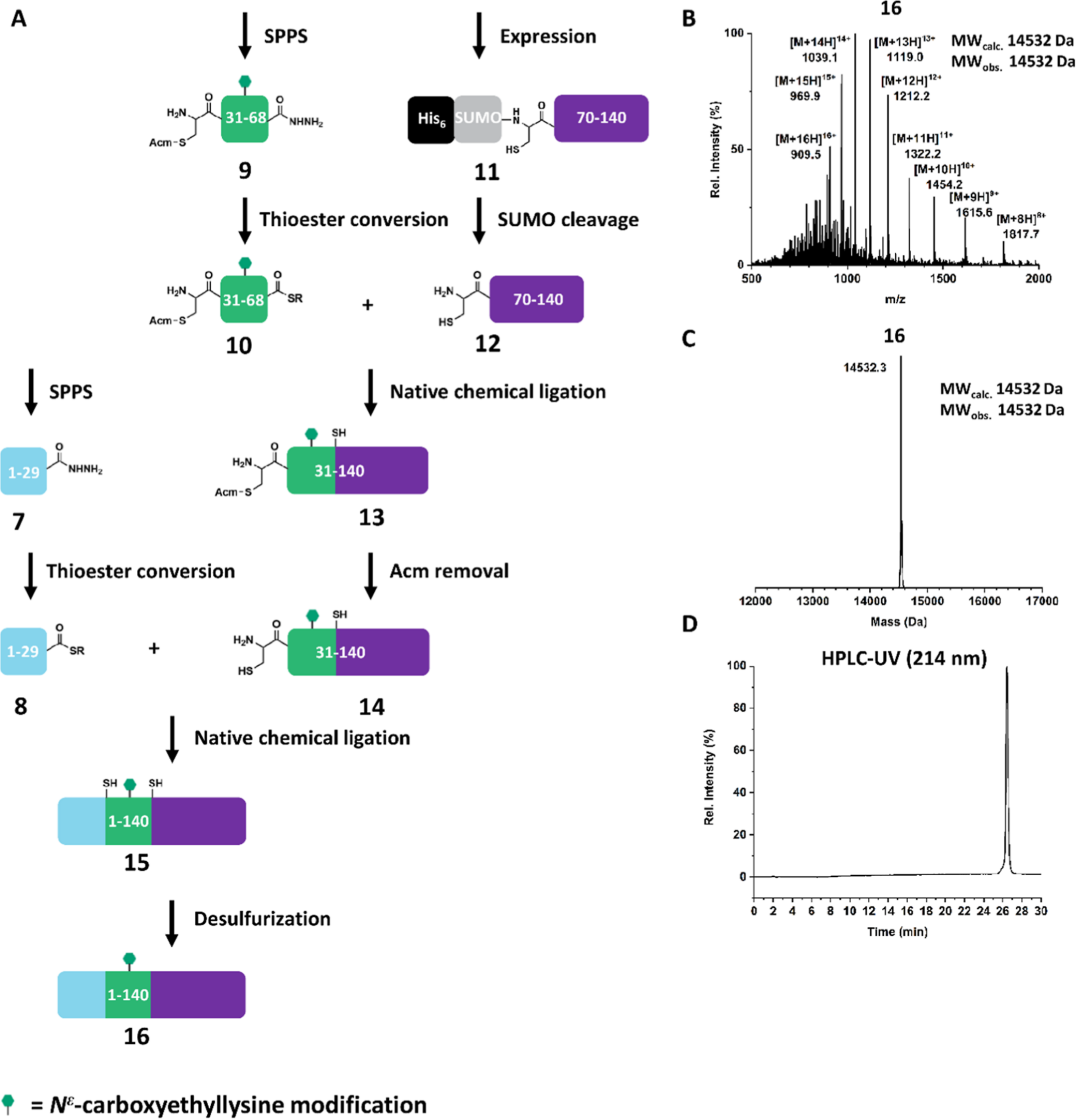
Three-segment ligation approach of αSyn. (A) Schematic overview of the semisynthetic strategy to generate CEL-modified αSyn at positions 32, 34, 43, 45, 58, and 60. (B) Mass spectrum and (C) deconvoluted spectrum of CEL-modified full-length αSyn **16**. (D) Purity of **16** was determined by HPLC-UV at 214 nm absorbance.

CEL incorporation in central segment C­(Acm)-αSyn 31-68 peptide hydrazide **9** was verified by MS (Figure S3D). The yields were 19%–35% for different CEL-modified variants, which was comparable to the reported 21% yield for the αSyn 30-68 A30C peptide hydrazide.^33^ Peptide hydrazide **9** was converted to MESNa thioester **10** by our standard protocol (Figure S3E) with yields of 59%–75% and high purity (Figure S3F).

Remaining C-terminal part **11** was recombinantly expressed as fusion construct His_6_-SUMO-αSyn 69-140 A69C and purified by Ni-NTA affinity chromatography (Figure S3G). Expression as a His_6_-TEV fusion construct was initially attempted, but did not yield detectable protein, likely due to the small size of the target fragment. Hence, the His_6_-SUMO tag was employed to enhance construct size and solubility. The isolated yield of **11** was 118 mg per liter of expression medium. Subsequently, a modified SUMO protease^38^ was used to remove the His_6_-SUMO tag in a traceless manner to generate **12** (αSyn 69-140 A69C) with 89% yield. Identity and purity of **12** were verified by MS and HPLC-UV (Figure S3H,I).

In a first NCL step, thioester **10** was ligated with **12** to form CEL-modified ligation product **13** (Figure S3J). The reaction was performed using our standard NCL protocol with MPAA as an additive and a reaction time of 16 h. Isolated yields for this step were 61%–73%. Before the second NCL reaction, the Acm protecting group was efficiently removed (61%–83% yield) by treatment with PdCl_2_ as described in a protocol from the Brik lab,^39^ and **14** was formed (Figure S3K). Intermediate **14** was ligated with thioester **8** analogous to the ligation in the two-segment semisynthesis. CEL-modified second ligation product **15** (αSyn 1-140 A30, 69C) was obtained (Figure S3L) with 67%–73% yield, which was comparable to the 59%–69% observed in the two-segment ligation strategy. VA-044 and TCEP were used for radical-mediated desulfurization to obtain CEL-modified αSyn **16** (Figure 3B,C). Similar to the two-segment synthesis, conversion during desulfurization was quantitative with isolated yields of 60%–74%, and no impurities were detected by HPLC-UV at 214 nm (Figure 3D). The combined yields of ligations and desulfurizations were 19%–25%, which were lower than those for the two-segment ligation strategy, but still an improvement compared to a previously reported three-segment ligation of αSyn.^35^

We additionally synthesized αSyn A53T, which is an important point mutation in PD,^6^ to demonstrate the general applicability of the three-segment NCL strategy. Therefore, alanine 53 of the central segment C­(Acm)-αSyn 31-68 peptide hydrazide was exchanged with threonine. Subsequent reaction steps were carried out as described for αSyn CEL variants, and reaction yields were reproducibly 10%–20% lower in αSyn A53T compared to WT and CEL-modified variants. Moreover, VA-044 and TCEP were insufficient for final desulfurization, and an alternative method with NaBEt_4_ ^40^ was necessary to achieve complete desulfurization with 40% isolated yield (Figure S4). The semisynthetic αSyn A53T was used as a disease-linked reference in the following biophysical experiments.

### Modified αSyn Variants Show Reduced Interactions with Phospholipid Vesicles

The secondary structures of semisynthetic αSyn variants were analyzed by circular dichroism (CD) spectroscopy (Figures 4A and S5). CD spectra of intrinsically disordered αSyn WT and A53T were indistinguishable and displayed a characteristic random-coil signal with a minimum at ∼200 nm wavelength, as reported previously.^41^ Introducing a single CEL modification did not change the CD spectra of any of the 11 modified αSyn variants, as exemplified by αSyn K23CEL and K34CEL variants. This is in agreement with earlier reports, showing that αSyn modification by MGO does not alter the secondary structure.^42^

**Figure 4:**
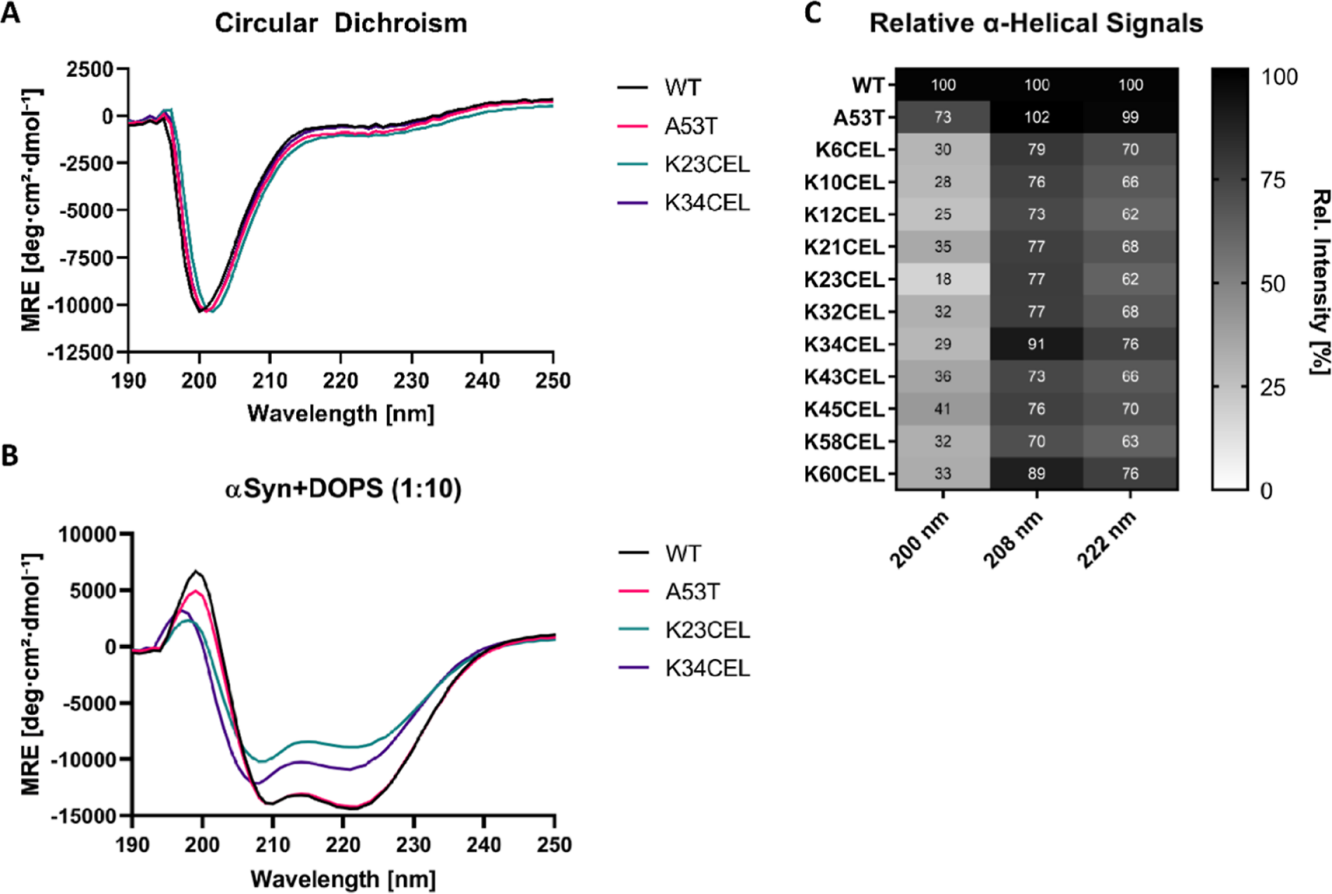
Structural characterization of αSyn variants by CD spectroscopy. (A) CD spectra of WT, A53T, and CEL-modified αSyn variants. (B) CD spectra of selected αSyn variants after addition of dioleoylphosphatidylserine (DOPS) liposomes. (C) Overview of relative CD signal intensities at α-helical wavelengths for all αSyn variants after DOPS addition.

Binding of αSyn to anionic phospholipid vesicles reportedly causes a conformational transition of the *N*-terminal region toward α-helical structures.^31^ Accordingly, addition of anionic dioleoylphosphatidylserine (DOPS) liposomes resulted in an increase of α-helical content, which was visible as a positive signal near 200 nm and characteristic minima at 208 and 222 nm in the CD spectra (Figures 4B and S5). At a 10-fold molar excess of DOPS, this conformational shift was comparable for αSyn WT and A53T, whereas the membrane-induced change in helicity was substantially lower in CEL-modified variants. Interactions of αSyn with membranes are primarily mediated by electrostatic interactions between positively charged lysine residues in the *N*-terminal region and negatively charged phospholipids.^43^ CEL formation converts lysine from a positively charged to a negatively charged residue, reversing electrostatic contributions and providing a mechanistic explanation for reduced conformational change. Although CD spectroscopy does not directly quantify binding affinity, the extent of induced α-helicity is widely used as a proxy for αSyn association with anionic lipid vesicles.^43^ Comparison of the relative signal intensities in CD spectra indicated a generally lower association of CEL-modified variants with anionic vesicles compared to αSyn WT (Figure 4C). Reduction of the 222 nm amplitude was highest for K23CEL and lowest for αSyn K34CEL with 38% and 24%, respectively. Consistent with our observations, previous studies have reported that heterogeneous glycation of αSyn by MGO impairs binding to anionic lipid vesicles.^26^ In summary, these data suggest that glycation of αSyn influences membrane interactions, which will require further validation for multiple CEL modifications and analysis in biological systems to assess pathological relevance.

### CEL Modification Alters αSyn Aggregation

Early oligomerization events of αSyn variants were monitored by dynamic light scattering (DLS) (Figure 5A). The hydrodynamic radius of αSyn WT increased from 5 to 774 nm over 48 h, whereas αSyn A53T oligomerized much faster and reached the upper detection limit of the Zetasizer Nano ZS at 2780 nm within the same time. In contrast, CEL-modified αSyn variants exhibited markedly slower oligomerization kinetics and formed substantially smaller assemblies with hydrodynamic radii ranging from 69 nm for αSyn K23CEL to 192 nm for αSyn K12CEL after 48 h (Figure 5B). At this time point, oligomers of αSyn WT were approximately 5–10-fold larger, while those of αSyn A53T were at least 15-fold larger than CEL-modified variants. Glycated αSyn displayed a higher initial hydrodynamic radius of 7–10 nm, compared to 3–5 nm reported for monomeric αSyn WT, and hydrodynamic radii in this range have been associated with αSyn tetramers or octamers previously.^44^ These observations indicate that single CEL modification favors the formation and stabilization of relatively small αSyn oligomers.

**Figure 5:**
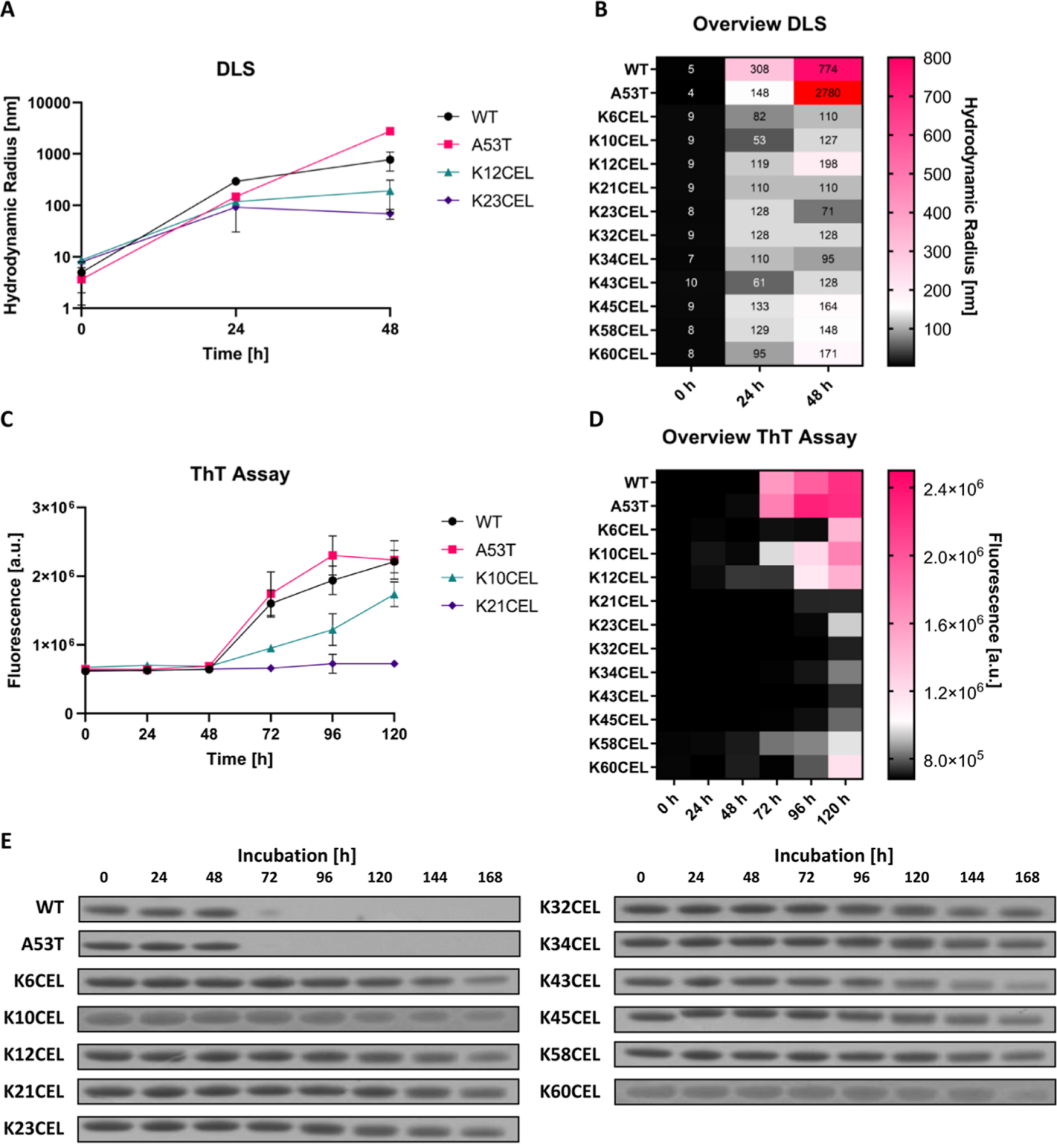
Aggregation behavior of αSyn variants. (A) DLS measurements illustrating early oligomerization behavior of selected αSyn variants (*n* = 3). (B) Overview of oligomerization for all analyzed αSyn variants obtained by DLS. (C) Aggregation kinetics of selected αSyn variants monitored by the thioflavin T (ThT) fluorescence assay (*n* = 5). (D) Overview of aggregation kinetics for all αSyn variants quantified by ThT fluorescence. (E) Representative images from sedimentation assays of selected αSyn variants.

Further progression of aggregation was assessed by ThT assays (Figure 5C), which indicate the formation of β-sheet-rich aggregates.^32^ A pronounced 2-fold increase in ThT fluorescence was detected for αSyn WT and A53T after incubation for 72 h, indicating the formation of fibrillar β-sheet structures. In contrast, CEL-modified variants exhibited substantially delayed or even attenuated ThT responses. For example, αSyn K10CEL required approximately 120 h to reach a 2-fold increase in ThT fluorescence, whereas αSyn K21CEL showed a negligible ThT fluorescence increase throughout the incubation period of 120 h. Comparison of aggregation kinetics across all CEL-modified variants revealed a reproducible position-dependent trend (Figure 5D). Modifications at *N*-terminal positions (K6CEL, K10CEL, and K12CEL) resulted in delayed aggregation onset at approximately 96 h, whereas variants modified at more proximal positions displayed little to no aggregation within 120 h.

To independently measure fibril formation, insoluble aggregate formation was examined by sedimentation assays, in which remaining soluble αSyn in supernatants was analyzed following centrifugation (Figures 5E and S6). No soluble αSyn was detectable by SDS–PAGE in αSyn WT and A53T samples after 72 h of incubation. Contrary to this, all CEL-modified αSyn variants retained detectable soluble protein even after 168 h, indicating a pronounced inhibition of fibril formation.

Aggregate morphology was further evaluated by scanning electron microscopy (SEM) (Figure 6). Samples of αSyn WT and A53T contained highly abundant, well-defined fibrillar structures, whereas aggregates were almost absent in samples containing CEL-modified αSyn variants. Additionally, glycated αSyn significantly differed in aggregate morphology from WT and A53T. Glycation resulted in much shorter fibrils, leading to predominantly diffuse, sponge-like aggregates. In the case of αSyn K58CEL, small, spherical particles were formed (Figure 6).

**Figure 6:**
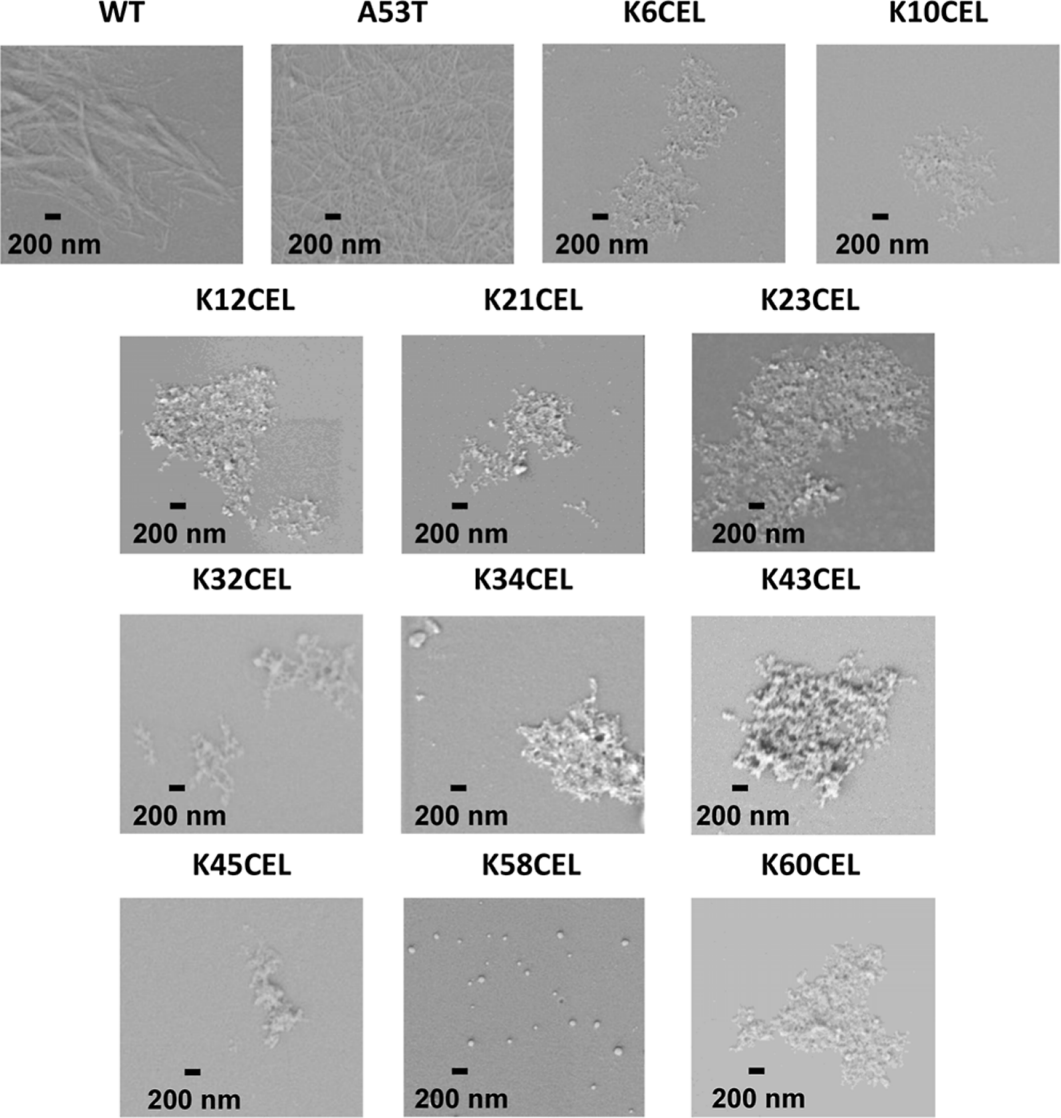
SEM of aggregated αSyn variants. Representative images of aggregated αSyn variants recorded by SEM.

Taken together, our data suggest a mechanistic link between CEL modification and previous observations made with heterogeneously glycated αSyn. Earlier studies demonstrated that MGO-induced glycation of recombinant αSyn promotes oligomer formation and inhibits fibrillization.^27^ Notably, our results show that even a single, site-specific CEL modification within the *N*-terminal region is sufficient to induce similar effects, comparable to extensive glycation of all lysine residues reported previously.^45^ Furthermore, aggregates formed by αSyn K58CEL closely resembled spherical structures such as reported for MGO-treated αSyn.^27^ Likewise, the diffuse morphologies observed for CEL-modified αSyn variants are consistent with aggregates formed upon ribose and glucose modification.^42^ Together, these observations underscore the pronounced impact of CEL modification on αSyn aggregation pathways. It remains to be explored how combinations of such modifications behave and which effects are most dominant.

### Seeding Activities of Modified αSyn

Beyond the effects described above, PTMs influence the ability of αSyn aggregates to promote further aggregation of monomeric αSyn through seeding processes.^46^ To assess this aspect, aggregation of αSyn WT was initiated by addition of 10% preformed aggregates derived from different αSyn variants, and aggregation kinetics were monitored by ThT assays (Figure 7A). Incubations containing only monomeric αSyn required approximately 48 h until aggregation. Seeding with aggregates of αSyn WT or A53T markedly reduced the lag phase, and the onset of aggregation was already detected after 12 h. In contrast, CEL-modified αSyn aggregates displayed heterogeneous seeding behavior that depended on the modification site (Figure 7B). Most CEL-modified variants exhibited intermediate seeding capacities, accelerating aggregation relative to monomeric αSyn, but were less efficient than aggregated αSyn WT. For example, aggregates of αSyn K6CEL, K32CEL, and K43CEL induced aggregation within approximately 24 h. No detectable seeding effect was observed for αSyn K21CEL and K60CEL, for which aggregation kinetics were comparable to unseeded controls. Notably, αSyn K10CEL was the only CEL-modified variant that displayed seeding efficiency similar to αSyn WT and induced aggregation within 12 h. Interestingly, even neighboring K6CEL or K12CEL had lower seeding capacity compared to K10CEL (Figure 7B).

**Figure 7:**
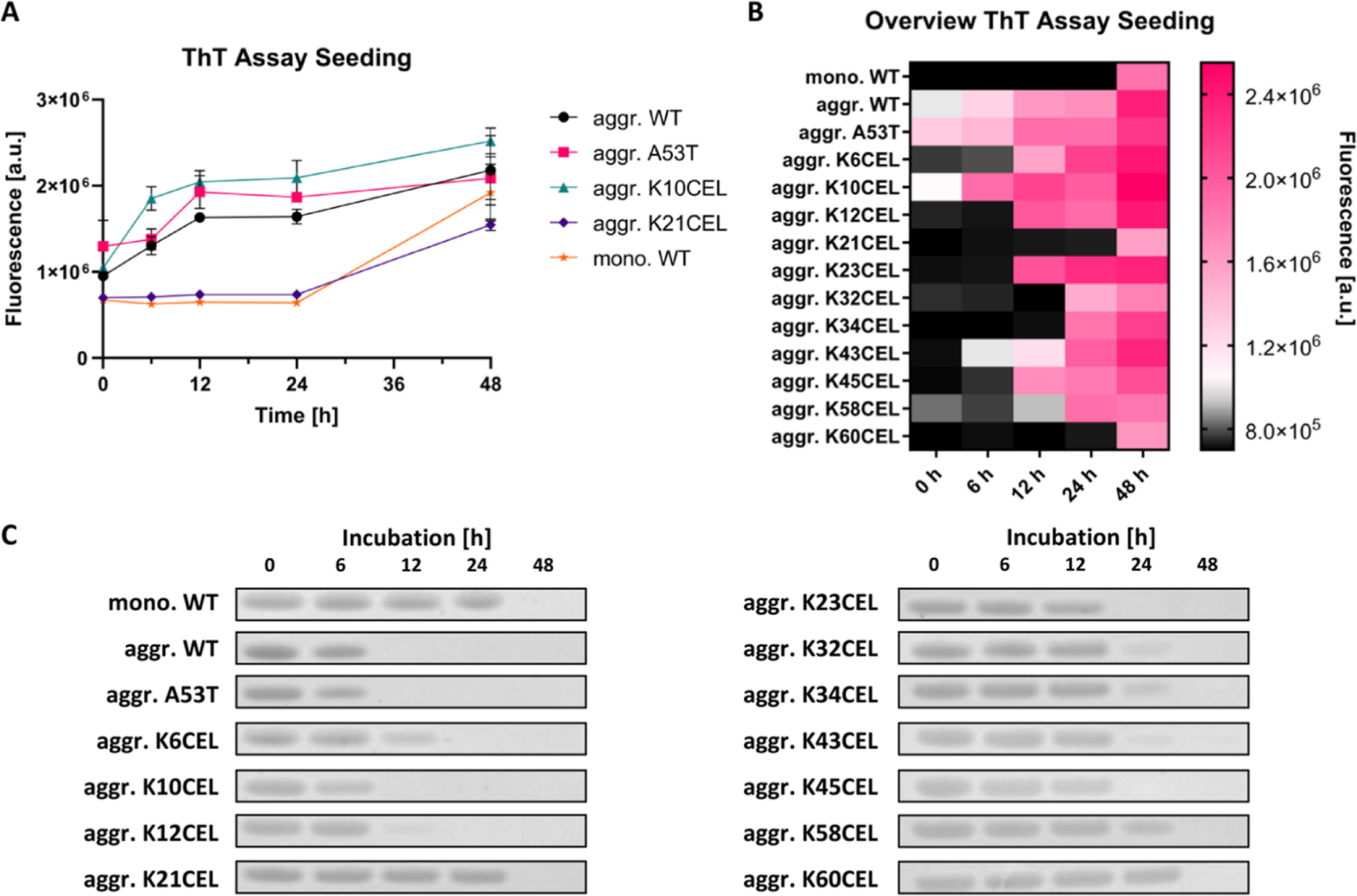
Seeding capacity of aggregated αSyn variants. (A) Aggregation kinetics of αSyn WT incubations seeded with 10% aggregated αSyn variants were monitored by the ThT assay (*n* = 3). (B) Overview of seeding effects by aggregated αSyn variants quantified by ThT fluorescence. (C) Representative images from the sedimentation assays of αSyn incubations seeded with aggregated αSyn variants.

To corroborate these findings, seeded incubations were analyzed using an orthogonal sedimentation assay to quantify the remaining soluble αSyn after incubation (Figures 7C and S7). Consistent with the ThT data, most CEL-modified variants showed intermediate depletion of soluble αSyn ranging between αSyn WT and monomeric controls. Seeds derived from αSyn K21CEL and K60CEL did not promote significant sedimentation, whereas variants K6CEL, K10CEL, and K12CEL showed substantial loss of soluble αSyn after 12 h, which was similar to effects observed for αSyn WT.

It is important to note that these in vitro seeding experiments are influenced by a complex interplay of factors, including the aggregation state and morphology of added seeds. For example, the low seeding activity observed for αSyn K21CEL may arise from impaired templating activity or from the reduced number of aggregates in the seeding material. Conversely, the strong seeding activity of αSyn K10CEL may be explained by its more advanced aggregated state. Moreover, the present experiments require further validation in cellular systems such as SH-SY5Y neuroblastoma cells or primary neurons as well as in vivo mice models in future studies for the final assessment of their pathological relevance. In this context, heterogeneous glycation of αSyn by MGO has previously been reported to enhance seeding in SH-SY5Y neuroblastoma cells,^47^ suggesting that additional cellular factors, such as uptake mechanisms or cellular localization (cytosolic vs membrane-bound state), may modulate the consequences of αSyn glycation.

## Conclusions

We established two semisynthetic strategies that enable site-specific incorporation of CEL-modified lysine residues into the *N*-terminal region of αSyn. These homogeneously modified αSyn variants allowed systematic dissection of single glycation effects and overcame key limitations of previous studies based on unspecific glycation by MGO.

We show that single CEL modifications do not alter the intrinsically disordered nature of αSyn, but markedly reduce membrane-induced α-helical folding. In addition, CEL incorporation stabilizes small αSyn oligomers, attenuates fibril formation, and induces changes in aggregate morphology. Seeding experiments revealed site-dependent effects, with most CEL-modified variants displaying reduced seeding capacity compared to αSyn WT, whereas K10CEL modification preserved efficient seeding activity.

Most importantly, our findings indicate that even single modifications by glycation, which are enriched in PD brain samples,^24^ have effects on αSyn behavior in vitro. The semisynthetic platform presented here provides a framework for future investigations in cellular and in vivo systems to assess the biological relevance of glycation to neurodegenerative diseases.

## Methods

### Chemicals and Materials

Fmoc (9-fluorenylmethyloxycarbonyl)-protected amino acids and pseudoproline dipeptides for SPPS were purchased from Iris Biotech (Marktredwitz, Germany). 2-Chlorotrityl chloride (2-CTC) polystyrene resin was purchased from Chemimpex (Illinois, USA). Unless otherwise stated, all additional chemicals and solvents were obtained from Sigma-Aldrich (Taufkirchen, Germany). Deionized water (Milli-Q reference A+) was used for all buffer preparations and experiments.

TEV protease was expressed in *E. coli* and purified as described previously.^48^ The SUMO protease expression plasmid pCDB327 was a gift from Christopher Bahl (Addgene plasmid #113671; http://n2t.net/addgene:113671; RRID:Addgene_113671) and was expressed as described previously.^38^

### Preparative HPLC

Preparative RP-HPLC was performed on a Varian ProStar system (Solvent Delivery Module, UV–vis Detector, Alltech Column Heater model 631, Waters Fraction Collector). Separation of the analytes was achieved using a semipreparative PerfectSil 300 ODS C4 column (10 μm, 300 Å, 250 × 10 mm) with gradient elution at a flow rate of 5.0 mL/min. Buffer A consisted of 0.1% trifluoroacetic acid (TFA) in water and buffer B was 0.08% TFA in acetonitrile (ACN). Samples were desalted at 5% buffer B for 15 min. Elution was performed by increasing buffer B to 20% over 5 min, followed by a linear gradient from 20% to 65% over 30 min. Absorbance was monitored at 214 nm wavelength.

### Analytical HPLC

Analytical RP–HPLC was performed on a Vanquish Horizon HPLC system equipped with a Kromasil C4 analytical column (5 μm, 300 Å, 4.6 × 150 mm). The flow rate was 1.0 mL/min and buffers were the same as for preparative HPLC. Elution was performed with a gradient from 5% to 65% buffer B in 30 min. Absorbance was monitored at both 214 and 280 nm.

### Mass Spectrometry

Liquid chromatography MS (LC–MS) was conducted on a Waters Arc system (SQ Detector 2, Quaternary Solvent Manager-R, Sample Manager FTN-R, 2489 UV/vis Detector, Zspray ESI source) equipped with a XBridge C4 column (3.5 μm, 400 Å, 2.1 × 100 mm). Buffer A consisted of 0.05% TFA in water and buffer B was 0.05% TFA in ACN. Samples were eluted using a linear gradient from 5% to 65% buffer B over 7 min at a 0.5 mL/min flow rate. Mass spectra were acquired in positive ion mode across an *m*/*z* range of 400–2000 utilizing electrospray ionization (ESI).

Direct ESI–MS was performed on a Waters analytical system (2767 Sample Manager, 515 HPLC Pump, 2545 Binary Gradient Module, System Fluidics Organizer, 2489 UV/vis Detector, 3100 Mass Detector). Samples were injected without chromatographic separation using a 1:1 mixture of buffer A (0.05% TFA in water) and buffer B (0.05% TFA in ACN) at a 1.0 mL/min flow rate. Mass spectra were acquired in positive ion mode across an *m*/*z* range of 400–2000.

### Solid-Phase Peptide Synthesis

Peptides were synthesized on a 0.05 mmol scale by microwave-assisted SPPS using a Liberty PRIME peptide synthesizer (CEM, Kamp-Lintfort, Germany). 2-CTC resin was pre-loaded with a hydrazine linker and Fmoc-amino acids, as described previously.^34^ Coupling steps were performed with 0.25 M *N,N′*-diisopropylcarbodiimide (DIC) in DMF and 0.5 M OxymaPure in DMF. Coupling was carried out under microwave irradiation at 70 °C for 6 min. Double couplings were performed following the synthesis of the first 20 amino acids. Fmoc-deprotection was achieved using 20% piperidine in DMF under microwave irradiation at 60 °C for 1.5 min. Pseudoproline dipeptides were used at approximately every 10th residue to enhance coupling efficiency. CEL modifications were incorporated site-specifically by coupling Fmoc- *l* -CEL­(OtBu)­(Boc)–OH in place of Fmoc- *l* -Lys­(Boc)–OH.

Cleavage from the resin was performed by addition of 3 mL of cleavage solution (87.5% TFA, 5% triisopropylsilane (TIPS), 5% dimethyl sulfide, 2.5% water) and incubation for 3 h at RT on a rotator. Following cleavage, the reaction mixture was filtered, and the peptide was precipitated by addition of 27 mL of cold diethyl ether. The crude product was purified by preparative HPLC, as described above, and lyophilized.

### Synthesis of αSyn 1–29 Thioester

Fragment αSyn 1–29 peptide hydrazide **1** was synthesized by the SPPS protocol described above starting from pre-loaded Fmoc-alanine hydrazide 2-CTC resin (0.5 mmol/g). Synthesis on a 0.05 mmol scale yielded 35–67 mg of the purified product (23%–45% yield).

For the conversion of hydrazide **1** to the corresponding thioester **2**, 23 mg (7.5 μmol) of **1** was dissolved in 3.5 mL of 6 M GdnHCl buffer (pH 3.0) and cooled to −15 °C. Then, 1.2 mL of a freshly prepared NaNO_2_ solution (1 mg mL^–1^) was added dropwise, and the reaction was stirred at −15 °C for 15 min. Thereafter, 3.3 mL of sodium 2-mercaptoethanesulfonate (MESNa) in water (9.5 mg mL^–1^) was added, the pH was adjusted to 6.7, and the mixture was stirred for 30 min at RT. The crude product was purified by preparative HPLC, fractions were analyzed by LC–MS, and after lyophilization, 16–21 mg (71%–88% yield) of **2** was obtained.

### Synthesis of C­(Acm)-αSyn 31-68 Thioester

Fragment C­(Acm)-αSyn 31-68 peptide hydrazide **9** was synthesized by the SPPS protocol described above starting from preloaded Fmoc-glycine hydrazide 2-CTC resin (0.3 mmol/g). Synthesis on a 0.05 mmol scale yielded 39–72 mg of the purified product (19%–35% yield).

Conversion of 7.5 μmol of hydrazide **9** to the corresponding thioester **10** using the above protocol afforded 18–23 mg (59%–75% yield).

### Transformation of *E. coli* BL21­(DE3)

The sequence for His_6_-TEV-αSyn 30–140 fusion protein **3** was cloned into a pET-21a­(+) bacterial vector containing an ampicillin (AMP) resistance gene by Gene Universal (Delaware, USA). The sequence for His_6_-SUMO-αSyn 69–140 A69C fusion protein **11** was cloned into a pET-21a­(+) bacterial vector containing an AMP resistance gene by BioCat (Heidelberg, Germany). Full-length sequences of the fusion proteins are provided in the Supporting Information.

Competent *E. coli* BL21­(DE3) bacterial cells were transformed by plasmids. After thawing 200 μL of bacterial suspension on ice, 5 μL of plasmid solution (0.5 ng of plasmid DNA) was added, and the cells were incubated for 15 min on ice. The cells were then subjected to a heat shock for 90 s at 42 °C and cooled again for 2 min on ice. The cells were incubated in 1 mL of lysogeny broth (LB) medium for 45 min at 37 °C. After centrifugation at 4000 RCF for 5 min, the supernatant was removed, and the pellet was resuspended in 200 μL of LB medium. Transformed *E. coli* cells were then plated onto an LB-agar plate (+100 μg/mL AMP) and incubated overnight at 37 °C. A single colony was used to inoculate 0.75 mL of LB medium (+100 μg/mL AMP) and grown at 37 °C for 6 h. Glycerol stocks were prepared by adding 0.75 mL of 40% glycerol and stored at −80 °C for later use.

### Expression of αSyn 30–140 A30C

A glycerol stock of *E. coli BL21­(DE3)* cells transformed with pET-21a­(+) His_6_-TEV-αSyn 30–140 plasmid was used to inoculate 600 mL of double-strength yeast extract tryptone (2YT) medium (+100 μg/mL AMP) for expression of fusion protein **3**. The inoculated medium was incubated at 37 °C overnight. The overnight culture was used to inoculate 6 L 2YT medium to an OD_600_ of 0.3. The bacterial culture was incubated until an OD_600_ of 0.7 was reached, and the expression of the target protein was induced by addition of 1 mM isopropyl β- *d* -1-thiogalactopyranoside (IPTG). Expression was carried out at 37 °C for 4 h.

The cells were harvested by centrifugation at 10,000 RCF for 10 min. The cell pellet was disrupted in 200 mL of lysis buffer (0.3 M NaCl, 10 mM imidazole, 50 mM NaH_2_PO_4_, pH 8.0) by sonication (10 min, 15 s pulses with 45 s pauses) with a Branson Sonifier SFX250 (Brookfield, USA). The lysate was cleared by 30,000 RCF centrifugation at 4 °C for 15 min. For purification by Ni-NTA chromatography, the supernatant containing fusion protein **3** was incubated with 20 mL of Ni-NTA resin (50% slurry) at 4 °C and gentle agitation for 1 h. The resin was then transferred equally into four 20 mL syringes and washed with 5 × 10 mL of lysis buffer. Bound protein was eluted with 2 × 10 mL of elution buffer (0.3 M NaCl, 250 mM imidazole, 50 mM NaH_2_PO_4_, pH 8.0). The solution was desalted by buffer exchange using 10 kDa Amicon centrifugal filters. After lyophilization, 35 mg of **3** was isolated per liter of culture medium.

To remove the affinity tag and obtain fragment **4**, 70 mg (5.3 μmol) of **3** was dissolved in 20 mL of protease buffer (100 mM NaCl, 2 mM DTT, 50 mM Tris, pH 8.0), TEV protease (0.1 eq., 7 mg) was added, and the reaction was incubated at 4 °C overnight. Possible cysteine adducts were removed by incubation with 50 mM methoxyamine at 4 °C for additional 24 h. The crude cleavage product was purified by preparative HPLC, yielding 42 mg (3.6 μmol, 78%) of purified product **4**.

### Expression of αSyn 69–140 A69C

A glycerol stock of *E. coli BL21­(DE3)* cells transformed with the pET-21a­(+) His_6_-SUMO-αSyn 69–140 A69C plasmid was used for the expression of fusion protein **11**. Expression and purification were carried out as described for **3**, with the exception that expression was performed at 18 °C for 16 h. After purification and lyophilization, 118 mg of **11** was obtained per liter of culture medium.

To obtain fragment **12**, His_6_ and SUMO tags were removed by SUMO protease. For cleavage, 235 mg (11.3 μmol) of desalted and lyophilized compound **11** was dissolved in 20 mL of protease buffer (100 mM NaCl, 2 mM DTT, 50 mM Tris, pH 8.0). SUMO protease (0.01 eq., 2.35 mg) was added, and the reaction was incubated overnight at 4 °C. Possible cysteine adducts were removed by incubation with 50 mM methoxyamine at 4 °C for additional 24 h. The crude cleavage product was purified by preparative HPLC, yielding 77 mg (10.1 μmol, 89%) of purified product **12**.

### Two-Segment Ligation Strategy

Two-segment NCL was used for site-specific incorporation of CEL at αSyn positions 6, 10, 12, 21, and 23. For NCL, 11.5 mg (1 μmol) of αSyn C-terminal fragment **4** was dissolved in 1.0 mL of ligation buffer (50 mM tris­(2-carboxyethyl)­phosphine (TCEP), 200 mM mercaptophenylacetic acid (MPAA), 6 M GdnHCl, 200 mM NaH_2_PO_4_, pH 8.5), and 6.4 mg (2.0 μmol) thioester **2** was added. The pH was adjusted to 7.2, and the reaction was incubated at 37 °C for 16 h. Successful ligation was verified by LC–MS, and after purification by preparative HPLC, 8.6–10.1 mg (0.6–0.7 μmol, 59%–69% yield) of ligation product **5** was obtained.

Finally, metal-free desulfurization^37^ was used to convert cysteine 30 to native alanine. Up to 1 μmol of **5** was dissolved in 1 mL of desulfurization buffer (250 mM TCEP, 100 mM MESNa, 20 mM VA-044, 6 M GdnHCl, 200 mM NaH_2_PO_4_). The pH was adjusted to 7.1, and the reaction was incubated at 37 °C for 3 h. LC–MS was used to verify conversion, and wild-type αSyn and CEL-modified αSyn variants **6** were obtained with yields of 72%–81%.

### Three-Segment Ligation Strategy

Three-segment NCL was used for site-specific incorporation of CEL at αSyn positions 32, 34, 43, 45, 58, and 60. For the first ligation step, 15.2 mg (2 μmol) of C-terminal fragment **12** was dissolved in 1.0 mL of ligation buffer (50 mM TCEP, 200 mM MPAA, 6 M GdnHCl, 200 mM NaH_2_PO_4_, pH 8.5), and 7.5 mg (4.0 μmol) of Acm-protected thioester **10** was added. The pH was adjusted to 7.2, and the reaction was incubated at 37 °C for 16 h. Ligation was monitored by LC–MS, and after purification by preparative HPLC, 14.3–17.0 mg (61%–73% yield) of the first ligation product **13** was obtained.

Before performing the second ligation, the Acm-group was removed by dissolving up to 17.5 mg (1.5 μmol) of CEL-modified **13** in 750 μL of 6 M GdnHCl buffer (200 mM NaH_2_PO_4_, pH 8.5) containing 50 eq. of MgCl_2_ and 15 eq. of PdCl_2_.^39^ After incubation at 37 °C for 2 h, the reaction was quenched by the addition of 750 μL of 500 mM DTT. Resulting fragment **14** was purified by preparative HPLC in 61%–83% yield.

Ligation of **14** and **8** yielded the second ligation product **15**, which was subsequently desulfurized to CEL-modified αSyn variant **16**. Ligation and desulfurization conditions were the same as described in the previous chapter. Yields for the ligation step were 67%–73%, and desulfurization yields were 60%–74%.

The αSyn A53T variant was synthesized following the same procedure as described above, with the exception that desulfurization was performed according to an alternative protocol.^40^ Intermediate αSyn 1–140 A53T A30,69C (7.5 mg, 0.52 μmol) was dissolved in 1 mL of desulfurization buffer (0.1 M citrate, 6 M GdnHCl, and 0.1 M TCEP at pH 4.5), followed by addition of 15 μL of NaBEt4 solution (1 g/mL), and the reaction mixture was incubated at RT for 15 min. The crude product was purified by semipreparative HPLC to afford 2.9 mg (0.2 μmol, 40% yield) of αSyn A53T.

### CD Spectroscopy

CD spectroscopy was used to characterize structural properties of αSyn variants and structural changes by binding to liposomes. CD spectra were obtained on a JASCO J-815/150S spectropolarimeter (Pfungstadt, Germany) at 25 °C in the range of 200–250 nm in 1 nm steps. Samples were measured at 0.25 mg mL^–1^ (17 μM) in a quartz microcuvette (1 mm path length) with phosphate-buffered saline (PBS) as a blank. Binding to liposomes and formation of α-helical structures were initiated by addition of anionic dioleoylphosphatidylserine (DOPS) liposomes at an αSyn:DOPS ratio of 1:10. For each sample, three spectra were averaged and normalized.

### Characterization of αSyn Aggregation

Semisynthetic αSyn variants were dissolved in aggregation buffer (50 mM Tris, 150 mM NaCl, pH 7.4) at a concentration of 0.25 mg mL^–1^ (17 μM) in 2 mL Eppendorf tubes. Aggregation was induced by incubation on an orbital shaker (1000 rpm) at 37 °C. To monitor the aggregation process during incubation, samples were analyzed by DLS, ThT assay, and sedimentation assay.

#### DLS

Early oligomer formation was assessed by DLS using a Malvern Zetasizer Nano ZS (Malvern, UK) with 1 mL folded capillary cells. Each sample was measured in triplicate, and hydrodynamic radius was calculated by “protein size” settings.

#### ThT Assay

Aggregates rich in β-sheet structures were detected by ThT binding. Fluorescence measurements were performed with a Molecular Devices SpectraMax i3x plate reader (San Jose, USA) using excitation at 450 nm and emission at 485 nm. Reactions were carried out in black 96-well plates with a final concentration of 1 μM protein and 100 μM ThT in 50 mM glycine buffer (pH 8.0), following established protocols.^32^

#### Sedimentation Assay

Insoluble αSyn fibrils were sedimented by centrifugation at 16,400 RCF for 10 min. Remaining soluble protein in the supernatant was quantitated by SDS–PAGE, as described previously.^32^ In short, a 15 μL aliquot of the supernatant was mixed with 5 μL of 4× NuPAGE LDS sample buffer and loaded onto precast 12-well NuPAGE 4%–12% Bis-Tris Mini Protein Gels, and gels were stained with SimplyBlue SafeStain. The prestained protein standard Precision Plus Protein Kaleidoscope (Bio-Rad, Hercules, USA) was used as the molecular weight marker. SDS–PAGE and gel staining were performed according to protocols by the manufacturer Thermo Fisher Scientific (Waltham, USA).

### Morphology of αSyn Aggregates

The morphology of αSyn aggregates was determined by SEM using a Zeiss Supra 55 VP electron microscope (Jena, Germany) under high-vacuum mode at an accelerating voltage of 2 kV. Images were acquired using a secondary electron detector (SE2) to obtain high-resolution surface topography.

Samples were prepared by incubating αSyn variants at 2.0 mg mL^–1^ (140 μM) in PBS under agitation (180 rpm) at 37 °C for 168 h. An aliquot was diluted with H_2_O to a final concentration of 70 μM. 5 μL of the diluted samples were applied onto carbon coated copper grids for 3 min. Grids were then blotted with filter paper, washed twice with 5 μL of water, and dried by air. To enhance conductivity and image quality, samples were sputter-coated with a 2 nm layer of gold before imaging.

### Seeding Activity

Wild-type αSyn was dissolved in aggregation buffer, and after addition of 10% pre-aggregated αSyn variants, aggregation kinetics were measured by ThT and sedimentation assays, as described above.

## Supplementary Material



## Data Availability

The data that support the findings of this study are available from the corresponding authors upon reasonable request.

## Notes

Sequences of peptides and proteins used in this study, and additional analytical data, including MS data, HPLC-UV chromatograms, CD spectra, and complete SDS-PAGE gels (PDF)

## References

[1] Parkinson J. (2002). An essay on
the shaking palsy. 1817. J. Neuropsychiatry
Clin. Neurosci..

[2] Luo Y., Qiao L., Li M., Wen X., Zhang W., Li X. (2025). Global, regional, national epidemiology and trends of Parkinson’s
disease from 1990 to 2021: findings from the Global Burden of Disease
Study 2021. Front. Aging Neurosci..

[3] Dorsey E. R., Sherer T., Okun M. S., Bloem B. R. (2018). The Emerging
Evidence
of the Parkinson Pandemic. J. Parkinson’s
Dis..

[4] Burré J., Edwards R. H., Halliday G., Lang A. E., Lashuel H. A., Melki R., Murayama S., Outeiro T. F., Papa S. M., Stefanis L., Woerman A. L., Surmeier D. J., Kalia L. V., Takahashi R. (2024). Research Priorities
on the Role of α-Synuclein
in Parkinson’s Disease Pathogenesis. Mov. Disord..

[5] Antony P. M.
A., Diederich N. J., Krüger R., Balling R. (2013). The hallmarks of Parkinson’s
disease. FEBS J..

[6] Lashuel H. A., Overk C. R., Oueslati A., Masliah E. (2013). The many faces of α-synuclein:
from structure and toxicity to therapeutic target. Nat. Rev. Neurosci..

[7] Makasewicz K., Linse S., Sparr E. (2024). Interplay of α-synuclein with
Lipid Membranes: Cooperative Adsorption, Membrane Remodeling and Coaggregation. JACS Au.

[8] Georgieva E. R., Ramlall T. F., Borbat P. P., Freed J. H., Eliezer D. (2008). Membrane-bound
alpha-synuclein forms an extended helix: long-distance pulsed ESR
measurements using vesicles, bicelles, and rodlike micelles. J. Am. Chem. Soc..

[9] Gallardo J., Escalona-Noguero C., Sot B. (2020). Role of α-Synuclein Regions
in Nucleation and Elongation of Amyloid Fiber Assembly. ACS Chem. Neurosci..

[10] Rinauro D. J., Chiti F., Vendruscolo M., Limbocker R. (2024). Misfolded
protein oligomers: mechanisms of formation, cytotoxic effects, and
pharmacological approaches against protein misfolding diseases. Mol. Neurodegener..

[11] Cremades N., Cohen S. I. A., Deas E., Abramov A. Y., Chen A. Y., Orte A., Sandal M., Clarke R. W., Dunne P., Aprile F. A., Bertoncini C. W., Wood N. W., Knowles T. P. J., Dobson C. M., Klenerman D. (2012). Direct observation
of the interconversion
of normal and toxic forms of α-synuclein. Cell.

[12] Ottolini D., Calí T., Szabò I., Brini M. (2017). Alpha-synuclein at
the intracellular and the extracellular side: functional and dysfunctional
implications. Biol. Chem..

[13] Tysnes O.-B., Storstein A. (2017). Epidemiology of Parkinson’s
disease. J. Neural Transm..

[14] Belvisi D., Pellicciari R., Fabbrini A., Costanzo M., Pietracupa S., De Lucia M., Modugno N., Magrinelli F., Dallocchio C., Ercoli T., Terravecchia C., Nicoletti A., Solla P., Fabbrini G., Tinazzi M., Berardelli A., Defazio G. (2020). Risk factors of Parkinson disease:
Simultaneous assessment, interactions, and etiologic subtypes. Neurology.

[15] Han K., Kim B., Lee S. H., Kim M. K. (2023). A nationwide cohort
study on diabetes
severity and risk of Parkinson disease. npj
Parkinson’s Dis..

[16] Yu H., Sun T., He X., Wang Z., Zhao K., An J., Wen L., Li J.-Y., Li W., Feng J. (2022). Association between
Parkinson’s Disease and Diabetes Mellitus: From Epidemiology,
Pathophysiology and Prevention to Treatment. Aging Dis.

[17] Vistoli G., Maddis D. de, Cipak A., Zarkovic N., Carini M., Aldini G. (2013). Advanced glycoxidation and lipoxidation end products
(AGEs and ALEs): an overview of their mechanisms of formation. Free Radic. Res..

[18] Baldensperger T., Preissler M., Becker C. F. W. (2025). Non-enzymatic
posttranslational protein
modifications in protein aggregation and neurodegenerative diseases. RSC Chem. Biol..

[19] Schalkwijk C. G., Stehouwer C. D. A. (2020). Methylglyoxal, a Highly Reactive Dicarbonyl Compound,
in Diabetes, Its Vascular Complications, and Other Age-Related Diseases. Physiol. Rev..

[20] Rabbani N., Thornalley P. J. (2012). Methylglyoxal, glyoxalase 1 and the
dicarbonyl proteome. Amino Acids.

[21] Baldensperger T., Eggen M., Kappen J., Winterhalter P. R., Pfirrmann T., Glomb M. A. (2020). Comprehensive analysis
of posttranslational
protein modifications in aging of subcellular compartments. Sci. Rep..

[22] Glomb M. A., Monnier V. M. (1995). Mechanism of protein
modification by glyoxal and glycolaldehyde,
reactive intermediates of the Maillard reaction. J. Biol. Chem..

[23] Baldensperger T., Jost T., Zipprich A., Glomb M. A. (2018). Novel α-Oxoamide
Advanced-Glycation Endproducts within the N6-Carboxymethyl Lysine
and N6-Carboxyethyl Lysine Reaction Cascades. J. Agric. Food Chem..

[24] Vicente
Miranda H., Szego É. M., Oliveira L. M. A., Breda C., Darendelioglu E., de Oliveira R. M., Ferreira D. G., Gomes M. A., Rott R., Oliveira M., Munari F., Enguita F. J., Simões T., Rodrigues E. F., Heinrich M., Martins I. C., Zamolo I., Riess O., Cordeiro C., Ponces-Freire A., Lashuel H. A., Santos N. C., Lopes L. V., Xiang W., Jovin T. M., Penque D., Engelender S., Zweckstetter M., Klucken J., Giorgini F., Quintas A., Outeiro T. F. (2017). Glycation potentiates α-synuclein-associated
neurodegeneration in synucleinopathies. Brain.

[25] Chegão A., Guarda M., Alexandre B. M., Shvachiy L., Temido-Ferreira M., Marques-Morgado I., Fernandes Gomes B., Matthiesen R., Lopes L. V., Florindo P. R., Gomes R. A., Gomes-Alves P., Coelho J. E., Outeiro T. F., Vicente Miranda H. (2022). Glycation
modulates glutamatergic signaling and exacerbates Parkinson’s
disease-like phenotypes. npj Parkinson’s
Dis..

[26] Uceda A. B., Frau J., Vilanova B., Adrover M. (2022). Glycation of α-synuclein
hampers its binding to synaptic-like vesicles and its driving effect
on their fusion. Cell. Mol. Life Sci..

[27] Lee D., Park C. W., Paik S. R., Choi K. Y. (2009). The modification
of alpha-synuclein by dicarbonyl compounds inhibits its fibril-forming
process. Biochim. Biophys. Acta Gen. Subj..

[28] Henning C., Glomb M. A. (2016). Pathways of the
Maillard reaction under physiological
conditions. Glycocon. J..

[29] de
Oliveira R. M., Vicente Miranda H., Francelle L., Pinho R., Szegö E. ´.
M., Martinho R., Munari F., Lázaro D. F., Moniot S., Guerreiro P., Fonseca L., Marijanovic Z., Antas P., Gerhardt E., Enguita F. J., Fauvet B., Penque D., Pais T. F., Tong Q., Becker S., Kügler S., Lashuel H. A., Steegborn C., Zweckstetter M., Outeiro T. F. (2017). The mechanism of sirtuin 2-mediated exacerbation of
alpha-synuclein toxicity in models of Parkinson disease. PLoS Biol..

[30] Balana A. T., Mahul-Mellier A.-L., Nguyen B. A., Horvath M., Javed A., Hard E. R., Jasiqi Y., Singh P., Afrin S., Pedretti R., Singh V., Lee V. M.-Y., Luk K. C., Saelices L., Lashuel H. A., Pratt M. R. (2024). O-GlcNAc
forces
an α-synuclein amyloid strain with notably diminished seeding
and pathology. Nat. Chem. Biol..

[31] Hejjaoui M., Butterfield S., Fauvet B., Vercruysse F., Cui J., Dikiy I., Prudent M., Olschewski D., Zhang Y., Eliezer D., Lashuel H. A. (2012). Elucidating the
role of C-terminal post-translational modifications using protein
semisynthesis strategies: α-synuclein phosphorylation at tyrosine
125. J. Am. Chem. Soc..

[32] Burai R., Ait-Bouziad N., Chiki A., Lashuel H. A. (2015). Elucidating the
Role of Site-Specific Nitration of α-Synuclein in the Pathogenesis
of Parkinson’s Disease via Protein Semisynthesis and Mutagenesis. J. Am. Chem. Soc..

[33] Zheng J.-S., Tang S., Qi Y.-K., Wang Z.-P., Liu L. (2013). Chemical synthesis
of proteins using peptide hydrazides as thioester surrogates. Nat. Prot..

[34] Mukherjee S., Vogl D. P., Becker C. F. W. (2023). Site-Specific Glycation of Human
Heat Shock Protein (Hsp27) Enhances Its Chaperone Activity. ACS Chem. Biol..

[35] Gatzemeier L. M., Meyer F., Diederichsen U., Outeiro T. F. (2023). Chemical Synthesis
of Alpha-Synuclein Proteins via Solid-Phase Peptide Synthesis and
Native Chemical Ligation. Chemistry.

[36] Cistrone P. A., Bird M. J., Flood D. T., Silvestri A. P., Hintzen J. C. J., Thompson D. A., Dawson P. E. (2019). Native Chemical
Ligation of Peptides and Proteins. Curr. Protoc.
Chem. Biol..

[37] Wan Q., Danishefsky S. J. (2007). Free-radical-based,
specific desulfurization of cysteine:
a powerful advance in the synthesis of polypeptides and glycopolypeptides. Angew. Chem., Int. Ed..

[38] Lau Y.-T. K., Baytshtok V., Howard T. A., Fiala B. M., Johnson J. M., Carter L. P., Baker D., Lima C. D., Bahl C. D. (2018). Discovery
and engineering of enhanced SUMO protease enzymes. J. Biol. Chem..

[39] Maity S. K., Jbara M., Laps S., Brik A. (2016). Efficient
Palladium-Assisted
One-Pot Deprotection of (Acetamidomethyl)­Cysteine Following Native
Chemical Ligation and/or Desulfurization To Expedite Chemical Protein
Synthesis. Angew. Chem., Int. Ed..

[40] Sun Z., Ma W., Cao Y., Wei T., Mo X., Chow H. Y., Tan Y., Cheung C. H. P., Liu J., Lee H. K., Tse E. C. M., Liu H., Li X. (2022). Superfast desulfurization for protein
chemical synthesis and modification. Chem.

[41] Jo E., McLaurin J., Yip C. M., St George-Hyslop P., Fraser P. E. (2000). alpha-Synuclein membrane interactions
and lipid specificity. J. Biol. Chem..

[42] Farzadfard A., König A., Petersen S. V., Nielsen J., Vasili E., Dominguez-Meijide A., Buell A. K., Outeiro T. F., Otzen D. E. (2022). Glycation
modulates alpha-synuclein fibrillization kinetics: A sweet spot for
inhibition. J. Biol. Chem..

[43] Pirc K., Ulrih N. P. (2015). α-Synuclein interactions with
phospholipid model
membranes: Key roles for electrostatic interactions and lipid-bilayer
structure. Biochim. Biophys. Acta Gen. Subj..

[44] Dong C., Hoffmann M., Li X., Wang M., Garen C. R., Petersen N. O., Woodside M. T. (2018). Structural
characteristics and membrane
interactions of tandem α-synuclein oligomers. Sci. Rep..

[45] Mariño L., Ramis R., Casasnovas R., Ortega-Castro J., Vilanova B., Frau J., Adrover M. (2020). Unravelling
the effect
of N­(ε)-(carboxyethyl)­lysine on the conformation, dynamics and
aggregation propensity of α-synuclein. Chem. Sci..

[46] Otzen D. E., Gamon L. F., Hägglund P., Nielsen J., Pedersen J. N., Nybo T., Nowak J. S., Amstrup S. K., Pirhaghi M., Davies M. J. (2026). Nitrated products
formed on α-synuclein are preferentially
incorporated into oligomers but excluded from fibrils: A mechanism
for accumulation of neurotoxic species. Biochim.
Biophys. Acta Gen. Subj..

[47] Vasili E., König A., Al-Azzani M., Bosbach C., Gatzemeier L. M., Thom S., Chegão A., Vicente Miranda H., Steinem C., Erskine D., Outeiro T. F. (2025). Glycation of alpha-synuclein
enhances aggregation and neuroinflammatory responses. npj Parkinson’s Dis..

[48] Phan J., Zdanov A., Evdokimov A. G., Tropea J. E., Peters H. K., Kapust R. B., Li M., Wlodawer A., Waugh D. S. (2002). Structural
Basis for the Substrate Specificity of Tobacco Etch Virus Protease. J. Biol. Chem..

