# Supplementary material for: Effects of Site-Specific Glycation on α‑Synuclein
Source: ACS Chem Biol. 2026 Jun 18;21(7):1765–76. doi: 10.1021/acschembio.6c00322 (PMC13386469; doi:10.1021/acschembio.6c00322)
Supplement: Supplementary file 1 [file cb6c00322_si_001.pdf]

Supporting Information

Effects of Site-Specific Glycation on α-Synuclein

Tim Baldensperger[1,*], Anna Hampel[1], Christian F. W. Becker[1,*]

[1]Institute of Biological Chemistry, Faculty of Chemistry, University of Vienna, Währinger Straße 38, 1090 Vienna, Austria.

* Corresponding authors

Table of Contents

Amino Acid Sequences S2

Figure S1. Intermediates of two-segment ligation strategy of αSyn. S3

Figure S2. Final analysis of αSyn WT. S4

Figure S3. Intermediates of three-segment ligation strategy of αSyn. S5

Figure S4. Final analysis of αSyn A53T. S6

Figure S5. CD spectra of CEL-modified αSyn variants. S7

Figure S6. Full-length SDS-PAGE gels of αSyn aggregation. S8

Figure S7. Full-length SDS-PAGE gels of αSyn seeding. S9

Amino Acid Sequences

Peptide sequences obtained by solid-phase peptide synthesis (SPPS) are written in the conventional format H–X–$NHNH_2$. Recombinantly expressed protein constructs are written in single-letter amino acid code from N- to C-terminus without terminal group annotation.

αSyn 1-29 peptide hydrazide:

H-MDVFMKGLSKAKEGVATVAAAEKTKQGVAEA-$NHNH_2$

C(Acm)-αSyn 31-68 peptide hydrazide:

H-C(Acm)GKTKEGVLYVGSKTKEGVVHGVATVAEKTKEQVTNVGG-$NHNH_2$

His_6-TEV-αSyn 30-140

HHHHHHENLYFQCGKTKEGVLYVGSKTKEGVVHGVATVAEKTKEQVTNVGGAVVTG
VTAVAQKTVEGAGSIAAATGFVKKDQLGKNEEGAPQEGILEDMPVDPDNEAYEMPSEE
GYQDYEPEA

His_6-SUMO-αSyn 69-140 A69C

MGSSHHHHHHGSGLVPRGSASMSDSEVNQEAKPEVKPEVKPETHINLKVSDGSSEIFFKI
KKTTPLRRLMEAFAKRQGKEMDSLRFLYDGIRIQADQTPEDLDMEDNDIIEAHREQIGG
CVVTGVTAVAQKTVEGAGSIAAATGFVKKDQLGKNEEGAPQEGILEDMPVDPDNEAYE
MPSEEGYQDYEPEA

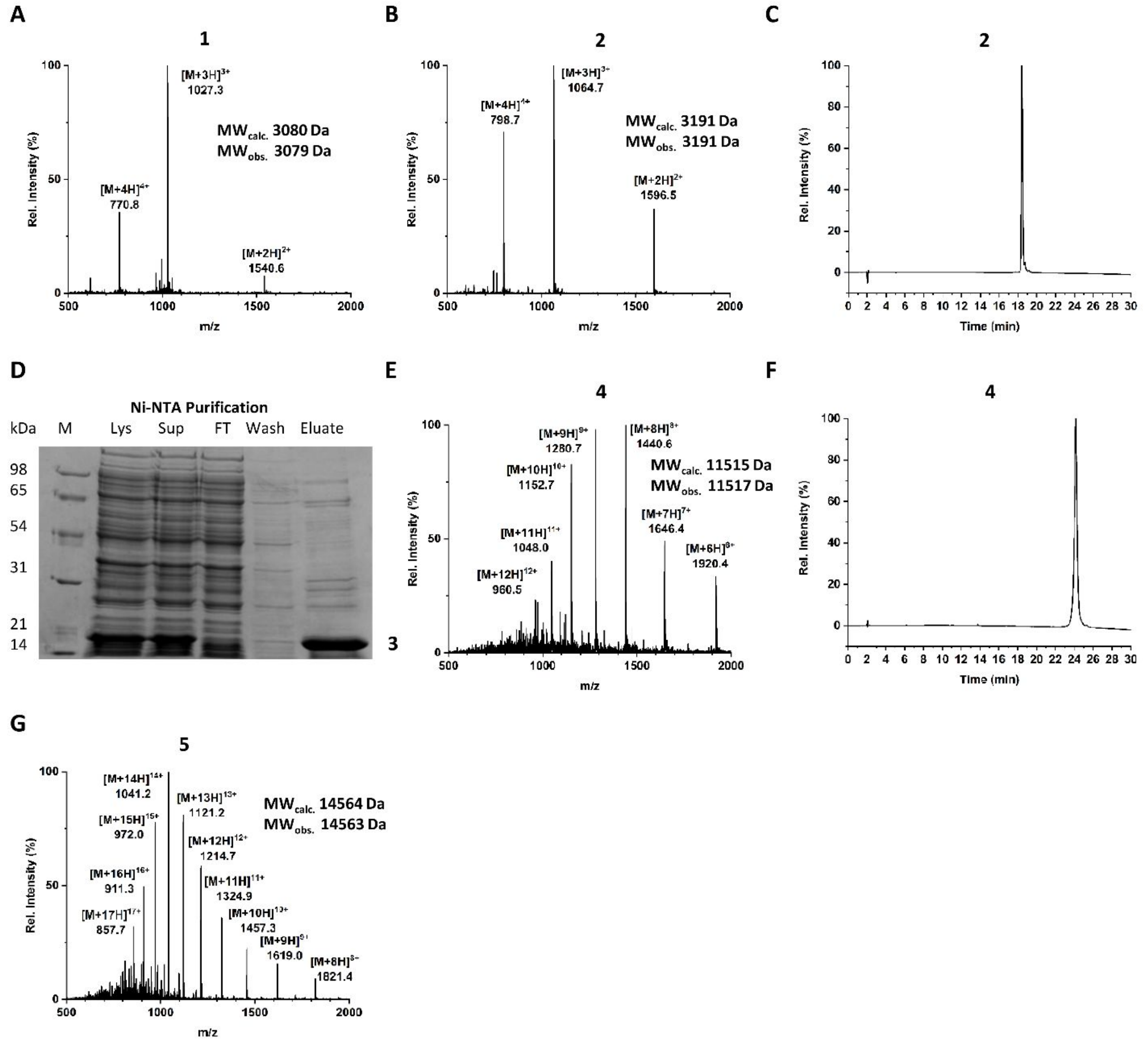

Figure S1. Intermediates of two-segment ligation strategy of αSyn. (A) Mass spectrum of CEL-modified αSyn 1-29 peptide hydrazide **1**. **(B)** Mass spectrum of CEL-modified αSyn 1-29 thioester **2**. **(C)** Purity of **2** was verified by HPLC-UV at 214 nm absorbance. **(D)** Purification of expressed His_6-TEV-αSyn 30-140 **3** by Ni-NTA affinity chromatography. Legend: M = Molecular weight marker, Lys = Lysate after cell disruption by sonication, Sup = Supernatant after centrifugation of lysate, FT = Flow-through of Ni-NTA column loading, Wash = Pooled fraction of 10 mM imidazole washing steps, Eluate = Elution with 250 mM imidazole. **(E)** Mass spectrum of TEV protease cleavage product αSyn 30-140 A30C **4**. **(F)** Purity of **4** was verified by HPLC-UV at 214 nm absorbance. **(G)** Mass spectrum of CEL-modified ligation product αSyn 1-140 A30C **5**.

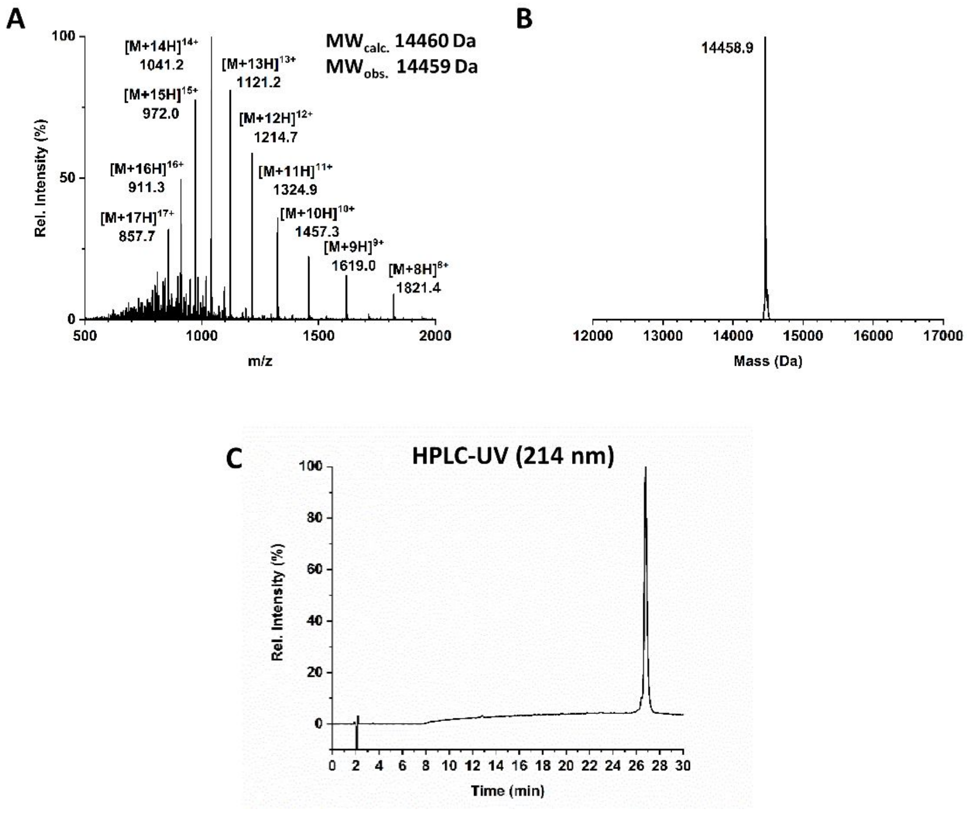

Figure S2. Final analysis of αSyn WT. (A) Mass spectrum, (**B)** deconvoluted spectrum, and **(C)** HPLC-UV chromatogram of αSyn WT.

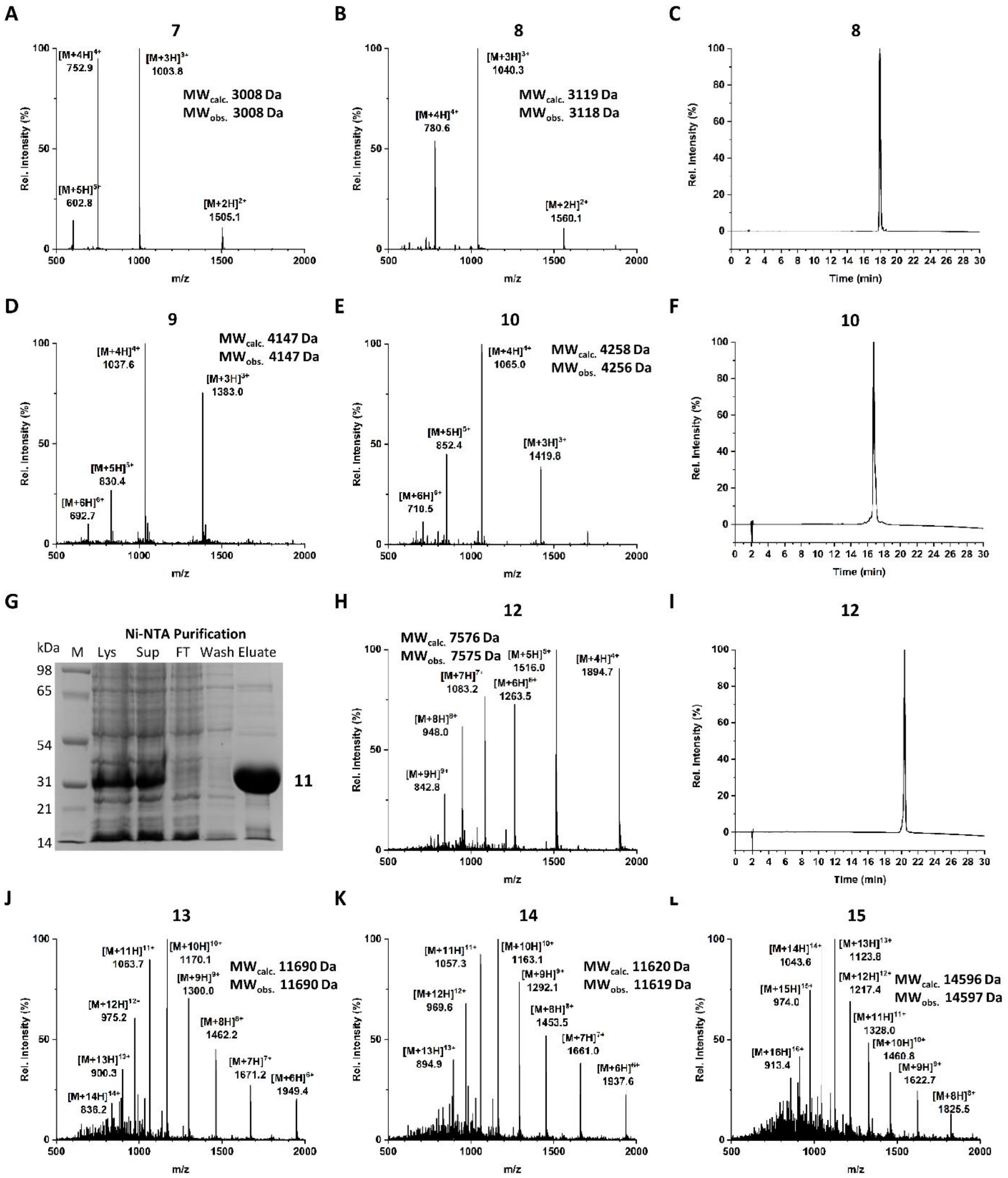

Figure S3. Intermediates of three-segment ligation strategy of αSyn. (A) Mass spectrum of αSyn 1-29 peptide hydrazide **7**. **(B)** Mass spectrum of αSyn 1-29 thioester **8**. **(C)** Purity of **8** was verified by HPLC-UV at 214 nm absorbance. **(D)** Mass spectrum of CEL-modified C(Acm)-αSyn 31-68 peptide hydrazide **9**. **(E)** Mass spectrum of CEL-modified C(Acm)-αSyn 31-68 thioester **10**. **(F)** Purity of **10** was verified by HPLC-UV at 214 nm absorbance. **(G)**

Purification of expressed His$_6$-SUMO-αSyn 69-140 A69C **11** by Ni-NTA affinity chromatography. Legend: M = Molecular weight marker, Lys = Lysate after cell disruption by sonication, Sup = Supernatant after centrifugation of lysate, FT = Flow-through of Ni-NTA column loading, Wash = Pooled fraction of 10 mM imidazole washing steps, Eluate = Elution with 250 mM imidazole. **(H)** Mass spectrum of SUMO protease cleavage product αSyn 69-140 A69C **12**. **(I)** Purity of **12** was verified by HPLC-UV at 214 nm absorbance. **(J)** Mass spectrum and spectrum of first CEL-modified ligation product C(Acm)-αSyn 31-140 A69C **13**. **(K)** Mass spectrum of first ligation product after Acm group removal **14**. **(L)** Mass spectrum of CEL-modified second ligation product αSyn 1-140 A30,69C **15**.

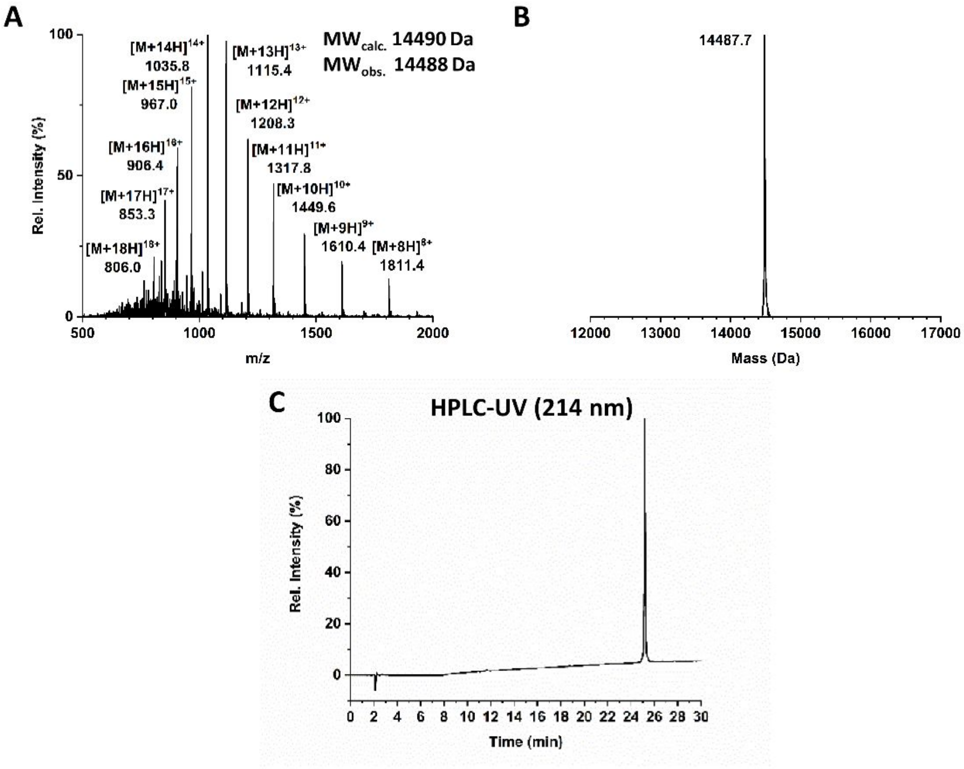

Figure S4. Final analysis of αSyn A53T. (A) Mass spectrum, (**B)** deconvoluted spectrum, and **(C)** HPLC-UV chromatogram of αSyn A53T.

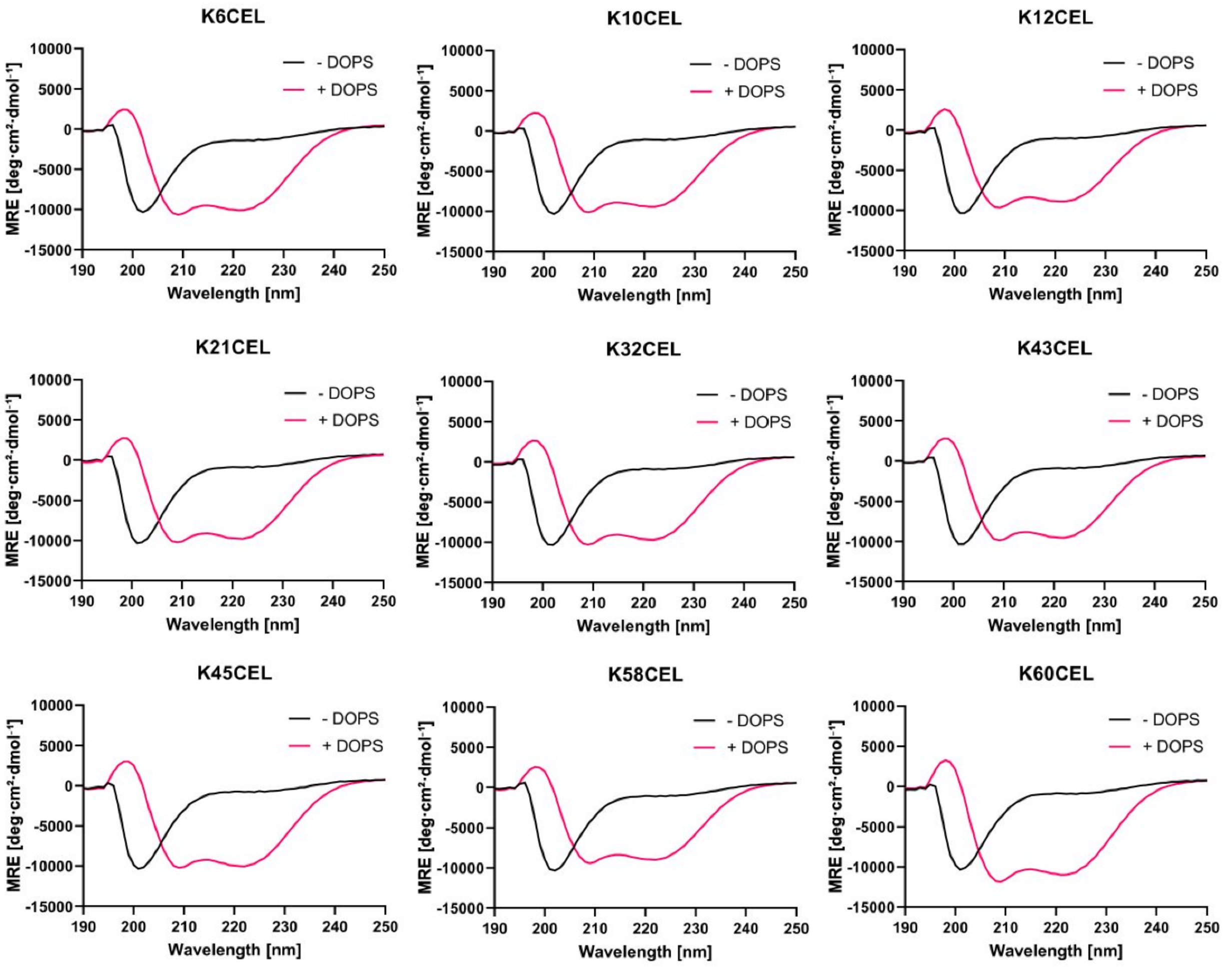

Figure S5. CD spectra of CEL-modified αSyn variants. The CD spectra of CEL-modified αSyn variants are shown in black and CD spectra after addition of DOPS are shown in magenta.

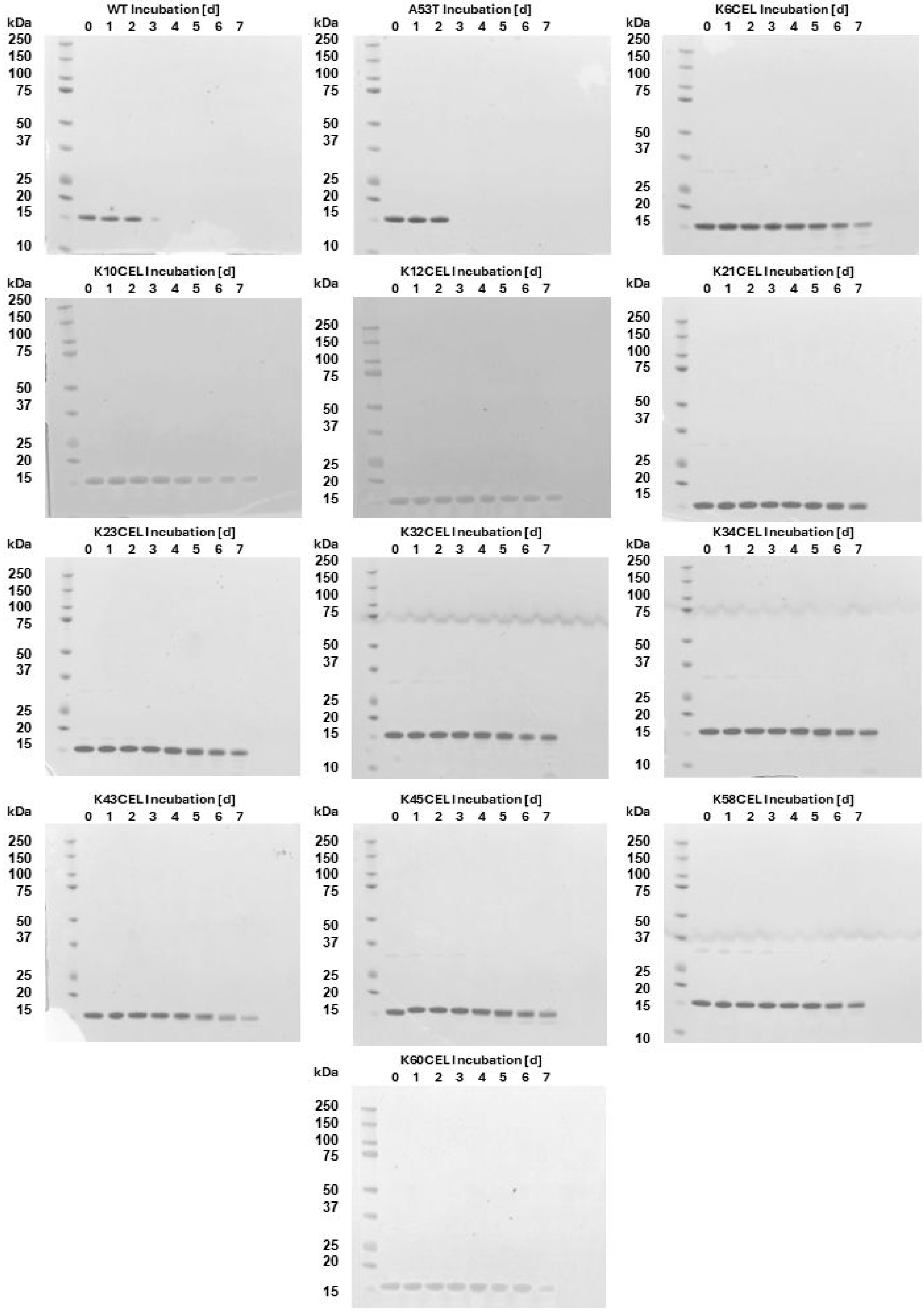

Figure S6. Full-length SDS-PAGE gels of αSyn aggregation.

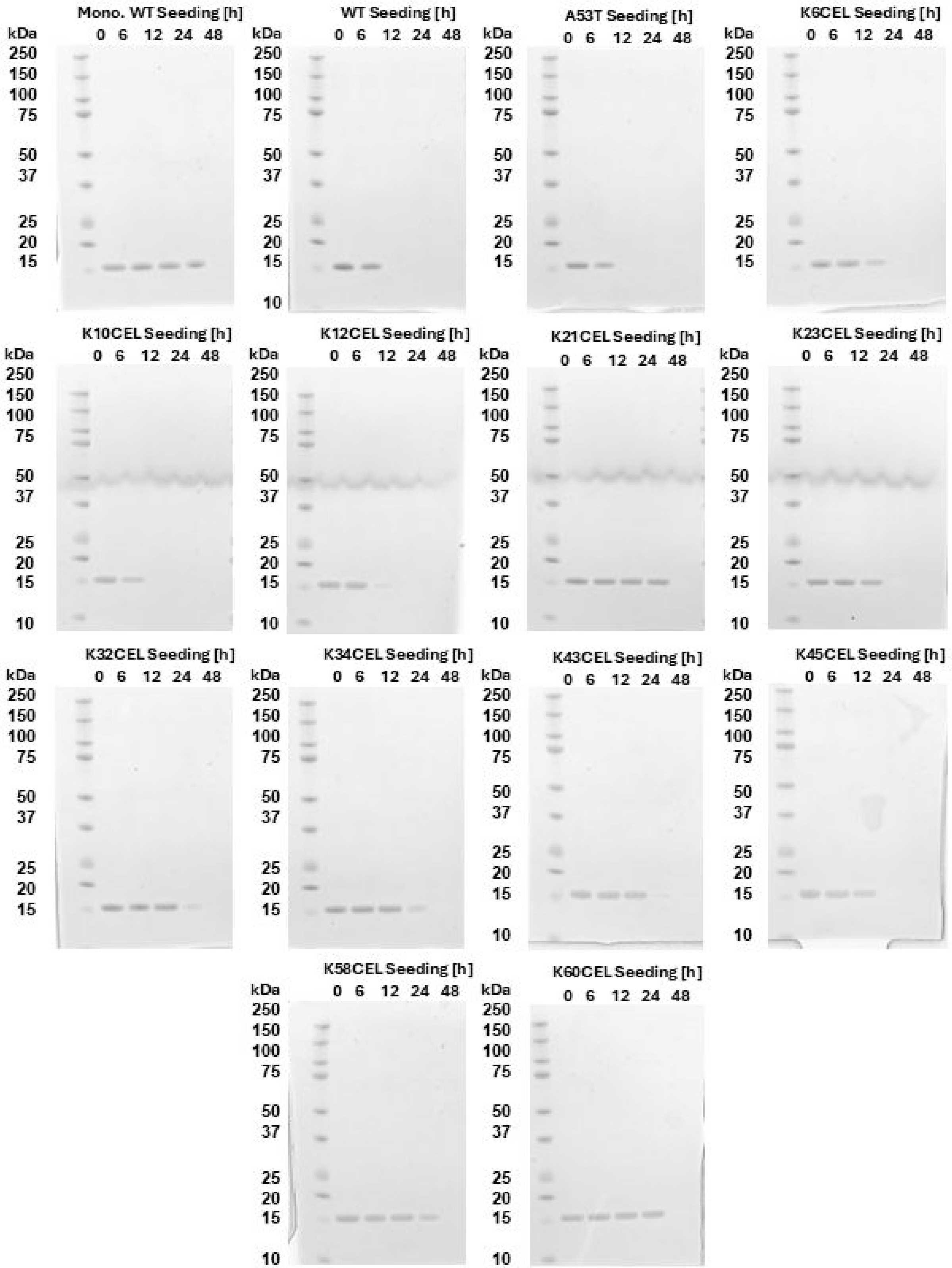

Figure S7. Full-length SDS-PAGE gels of αSyn seeding.
